# Rarity: discovering rare cell populations from single-cell imaging data

**DOI:** 10.1093/bioinformatics/btad750

**Published:** 2023-12-13

**Authors:** Kaspar Märtens, Michele Bortolomeazzi, Lucia Montorsi, Jo Spencer, Francesca Ciccarelli, Christopher Yau

**Affiliations:** The Alan Turing Institute, London NW1 2DB, United Kingdom; Francis Crick Institute, London NW1 1AT, United Kingdom; King’s College London, London WC2R 2LS, United Kingdom; Francis Crick Institute, London NW1 1AT, United Kingdom; King’s College London, London WC2R 2LS, United Kingdom; King’s College London, London WC2R 2LS, United Kingdom; Francis Crick Institute, London NW1 1AT, United Kingdom; Bart’s Cancer Institute - Centre for Cancer Genomics & Computational Biology, Queen Mary University of London, Charterhouse Square, London, EC1M 6BQ, United Kingdom; The Alan Turing Institute, London NW1 2DB, United Kingdom; Nuffield Department for Women’s & Reproductive Health, University of Oxford, Women’s Centre (Level 3), John Radcliffe Hospital, Oxford OX3 9DU, United Kingdom

## Abstract

**Motivation:**

Cell type identification plays an important role in the analysis and interpretation of single-cell data and can be carried out via supervised or unsupervised clustering approaches. Supervised methods are best suited where we can list all cell types and their respective marker genes *a priori*, while unsupervised clustering algorithms look for groups of cells with similar expression properties. This property permits the identification of both known and unknown cell populations, making unsupervised methods suitable for discovery. Success is dependent on the relative strength of the expression signature of each group as well as the number of cells. Rare cell types therefore present a particular challenge that is magnified when they are defined by differentially expressing a small number of genes.

**Results:**

Typical unsupervised approaches fail to identify such rare subpopulations, and these cells tend to be absorbed into more prevalent cell types. In order to balance these competing demands, we have developed a novel statistical framework for unsupervised clustering, named Rarity, that enables the discovery process for rare cell types to be more robust, consistent, and interpretable. We achieve this by devising a novel clustering method based on a Bayesian latent variable model in which we assign cells to inferred latent binary on/off expression profiles. This lets us achieve increased sensitivity to rare cell populations while also allowing us to control and interpret potential false positive discoveries. We systematically study the challenges associated with rare cell type identification and demonstrate the utility of Rarity on various IMC datasets.

**Availability and implementation:**

Implementation of Rarity together with examples is available from the Github repository (https://github.com/kasparmartens/rarity).

## Background

High-dimensional molecular analysis of single cells with highly multiplexed imaging allows the simultaneous measurement of the expression of multiple proteins while retaining information about their spatial origin within the tissue section. Technologies such as imaging mass cytometry (IMC) (Giesen *et al.* 2014) and multiplexed ion beam imaging (MIBI) (Angelo *et al.* 2014) use antibodies conjugated with heavy metals to stain tissues, which is followed by laser ablation and mass spectrometry to quantify expression of around 40 predetermined molecular markers. Immunofluorescence-based microscopy, such as multiplexed immunofluorescence (Gerdes *et al.* 2013) and cyclic immunofluorescence (CyCIF) (Lin *et al.* 2018), allows the multiplexed detection of proteins using standard microscopy.

A high-throughput single-cell analysis using such technologies can therefore lead to molecular profiles of tens of thousands of cells. For instance, Damond *et al.* (2019) used IMC to analyse and characterize the pathogenesis and progression of type 1 diabetes in human patients, while Bortolomeazzi *et al.* (2020) integrated multiregional whole-exome, RNA, and T cell receptor sequencing as well as IMC to examine the tumour microenvironment of hypermutated colorectal cancers in response to anti-PD1 immunotherapy. MIBI-TOF (Keren *et al.* 2018) was used to profile 36 immune-related proteins (including PD1, PD-L1, and IDO) in 41 triple-negative breast cancer patients to reveal mixed and compartmentalized tumours that coincided with cell type and location-specific expression of key markers.

A standard step in single-cell analysis is cell type identification and classification in which cells (data points) are sorted into phenotypically distinct groups (clusters). This can be accomplished via supervised (Abdelaal *et al.* 2019, Geuenich *et al.* 2021, Cui *et al.* 2023) or unsupervised (Levine *et al.* 2015, Van Gassen *et al.* 2015) clustering approaches. A number of computational packages are now available to simplify the use of such analysis (Eling *et al.* 2020, Opzoomer *et al.* 2021). However, typically clustering algorithms are not specifically designed for the exploration of *rare* cell populations. A review of 18 clustering methods in Weber and Robinson (2016), including FlowSOM (Van Gassen *et al.* 2015) and PhenoGraph (Levine *et al.* 2015), across six high-dimensional single-cell flow and mass cytometry data demonstrated a wide variation in performance in rare cell population detection.

**Figure 1.**
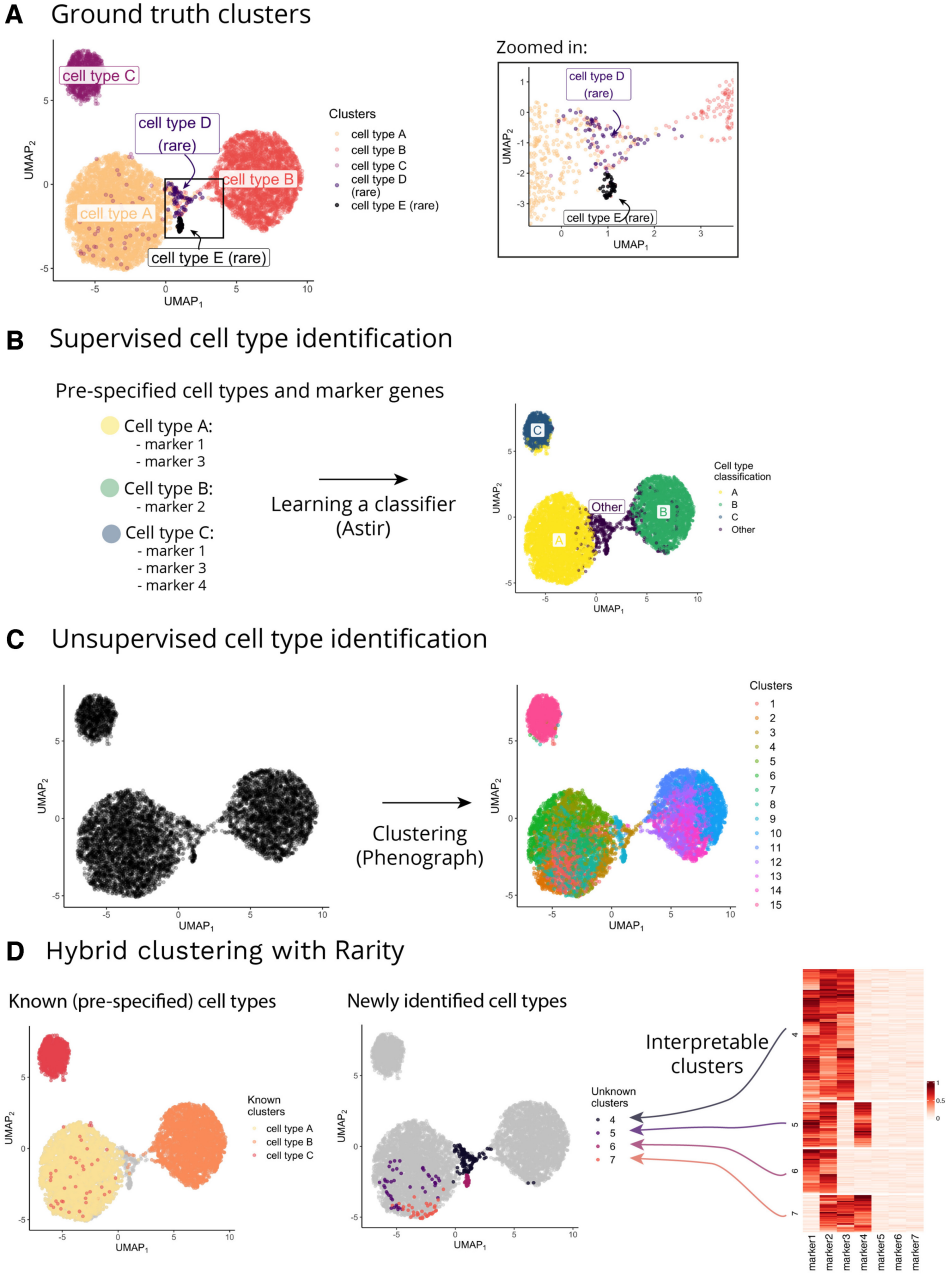
Comparison of supervised and unsupervised workflows for cell type identification on a synthetic dataset highlights their drawbacks motivating our hybrid modelling framework. (A) The synthetic dataset comprises five different cell populations (cell types (A–E)), two of which are rare and unknown to us a priori (cell types (D) and (E)). The UMAP plot is coloured by ground truth cell type labels. The zoomed-in panel shows a close-up of the two rare cell types (note that the UMAP visualization has failed to recognize cell type (D) as a distinct cluster). (B) In the supervised case (here shown for Astir (Geuenich *et al.* 2021)), our capabilities to detect cell types are limited to the prespecified cell types and their characteristic markers. The rare cell types are either merged with other known cell types or assigned to a separate ‘Other’ cluster. (C) In the unsupervised case, the workflow involves running a clustering algorithm (here shown for Phenograph), followed by manual inspection of marker genes in order to label (and potentially merge) the inferred clusters. (D) The proposed hybrid approach Rarity can help in identifying both prespecified cell types (as shown in the left UMAP) as well as rare novel cell populations (as shown in the right UMAP). The identified clusters are interpretable in terms of their differential expression profile—the identified rare clusters are shown in the heatmap.

Figure 1 illustrates the challenges using a synthetic dataset (see section ‘Methods’ for simulation details) containing three *known* and two *unknown* cell populations—the former (cell types A–C) are present at a high prevalence (49%, 33%, and 12% of cells, respectively), whereas the latter (cell types D–E) are rare (with prevalence below 1%). Figure 1A highlights how the Uniform Manifold Approximation and Projection (UMAP) dimensionality reduction has not recognized cell type D as a recognizably distinct cluster, instead it is mixed with the more prevalent cell types A and B. When applying a supervised model Astir (Geuenich *et al.* 2021) after specifying the marker genes for the known cell types A–C, Astir assigns both rare cell types to an ‘Other’ category (Fig. 1B) but does not provide functionality for any further analysis of these cells. When applying an unsupervised model Phenograph (Levine *et al.* 2015) (Fig. 1C), we identify 15 clusters, but the true rare cell type D is split across four clusters.

In this article, we describe *Rarity*, a hybrid semi-supervised framework for cell type identification that has been specifically developed to enable user-controlled sensitivity to rare subpopulations. We demonstrate that Rarity is able to identify putative rare cell populations that existing clustering methods do not identify and cannot be visualized with high-dimensional visualization techniques such as t-distributed stochastic neighbor embedding (t-SNE) (Van der Maaten and Hinton 2008) and UMAP (Becht *et al.* 2018, McInnes *et al.* 2018) (for further details, see Supplementary Section A). Further, through the use of a binary latent feature model, we illustrate how Rarity assigns a simple and interpretable binary marker signature to each cluster making post hoc examination, filtering, and verification of clusters substantially easier.

## Results

### Rarity: a hybrid clustering framework for detecting rare cell populations

**Figure 2.**
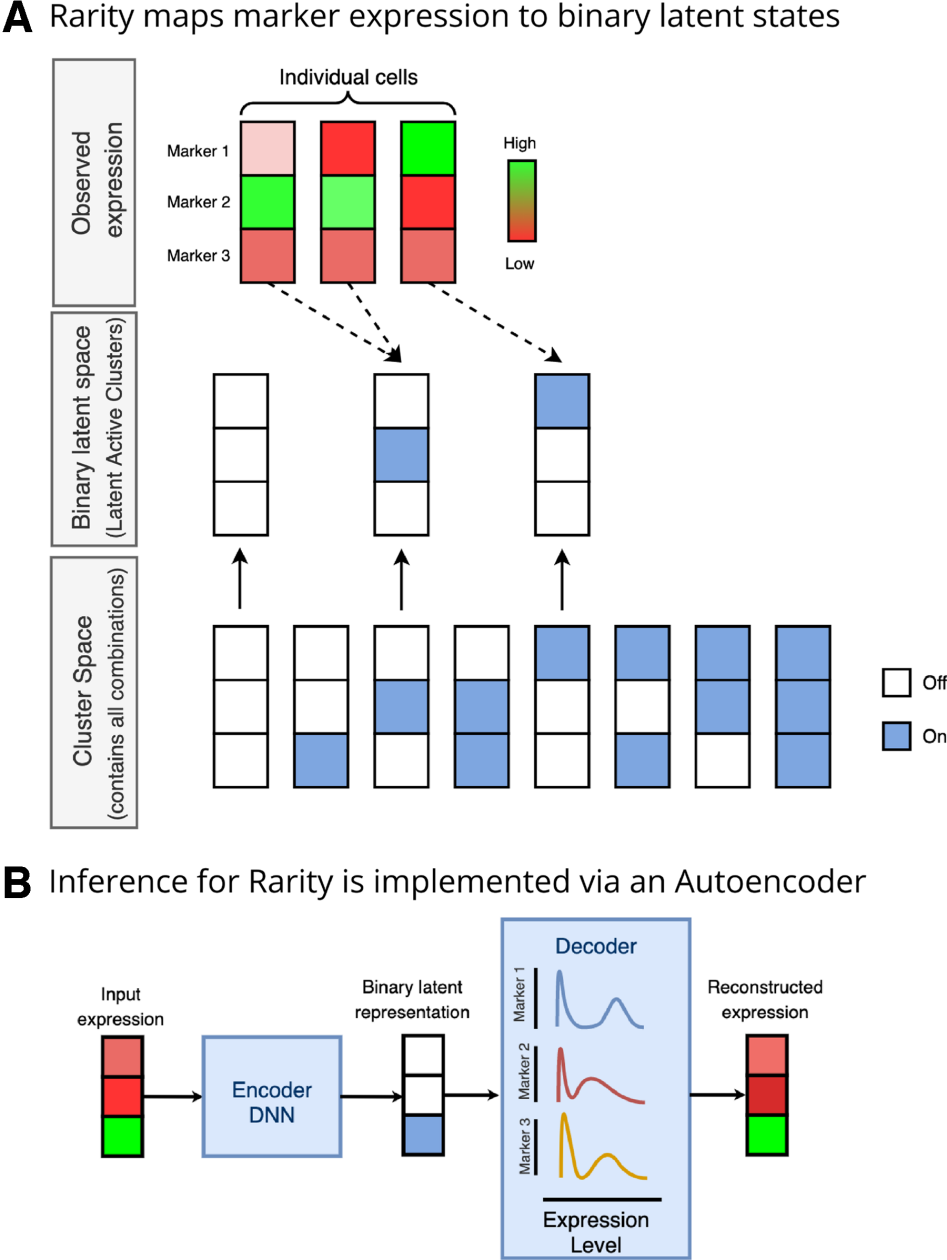
Overview of Rarity model specification and implementation. (A) Rarity projects single cell marker intensity vectors (illustrated for three markers and three cells, top panel) onto binary expression signatures (in the middle panel). Note that the active latent signatures cover only a subset of all possible binary combinations in the cluster space (in the bottom panel). (B) Tractable inference for Rarity is implemented as a structured autoencoder, where an encoder neural network is used in combination with continuous relaxations to project expression vectors to binary expression signatures.

Rarity combines the benefits of supervised and unsupervised approaches to cell type identification in a hybrid probabilistic framework and is designed to be sensitive to rare subpopulations, including those which differ from other cell types in the expression of even a single marker. To achieve this level of sensitivity without sacrificing interpretability, we condition upon a statistical modelling assumption—the continuous marker expression values associated with each cell have an underlying binary on/off state. We model these unobserved on/off states as binary latent variables. Every cell with the same binary signature across the features is then assigned to the same cluster (Fig. 2A). The cluster space contains $2^{P}$ possible clusters, where *P* is the number of features, which are each associated with one of the 2*P* possible combinations of on/off states across the *P* features. Known cell types can therefore be specified by *a priori* specifying the appropriate binary expression pattern.

Rarity is implemented within a variational autoencoder framework (Fig. 2B). As a result, our implementation also scales favourably to a large number of cells. We employ inference amortization, an approximate inference technique which introduces an encoder neural network as a form of parameter sharing (Kingma and Welling 2014, Rezende *et al.* 2014) across cells. As a result, even though the binary expression signatures are inferred for every cell individually, the number of learnable parameters is fixed and does not grow with the number of cells in our data. Moreover, having a trained model, we can employ it on new cells without additional training.

Code and use case examples for Rarity are available from a Github repository https://github.com/kasparmartens/rarity.

### Self-consistency clustering experimental procedures and metrics

To investigate the sensitivity of existing IMC clustering methods for detecting cell types present at various prevalence levels in a controlled setting, we use (semi-)synthetic simulation experiments with ground-truth labels. Our experiments involve applying a clustering algorithm to a simulated or real dataset to derive clusters corresponding to cell types. We then downsample one of the identified clusters to create a new dataset with fewer cells of that type and recluster using the same method to measure how similar (or different) the inferred clusters from the downsampled dataset will be from the original one. A desirable property of a clustering method is self-consistency, i.e. the ability to allocate cells in the same cluster regardless of the prevalence of each cluster (such as after downsampling).

To quantify the quality of the inferred clustering, we adopt two metrics that capture different aspects of self-consistency. First we measure homogeneity, i.e. the property of the inferred clusters to contain only a distinct cell type. Second, we measure completeness, i.e. the property of grouping all cells of a particular type in only one cluster (Rosenberg and Hirschberg 2007). Since our focus lies in the identification of rare cell types, in our downsampling experiments we measure these two scores conditional on the downsampled cluster. We refer to these conditional scores as conditional homogeneity and conditional completeness, both with values between 0 and 1, higher scores being better (see section ‘Methods’ for more details). To further summarize these two scores with a single summary statistic, we use the harmonic average of the two, the conditional V-measure (Rosenberg and Hirschberg 2007).

**Figure 3.**
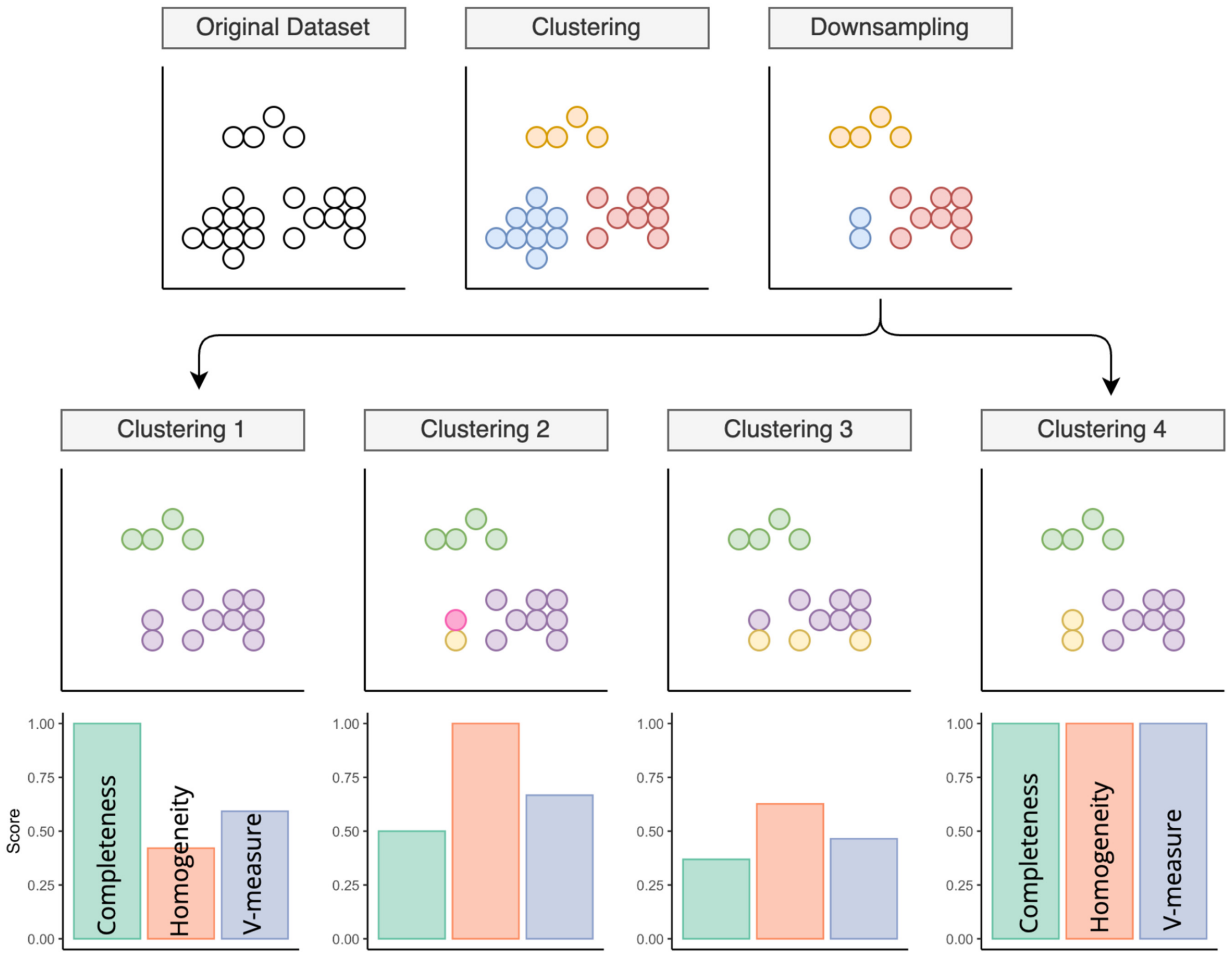
Downsampling simulation experiments and benchmark metrics to quantify self-consistency. Schematic showing how the downsampling experiments were conducted, and how different outcome scenarios affect the clustering performance metrics.

The behaviour of these metrics is illustrated in Fig. 3 for four different clustering scenarios (Clustering 1–4). Suppose a clustering method identifies three cell types (red, yellow and blue) from the original data. We then reduce the number of blue cells to create a downsampled dataset. In the first scenario (Clustering 1), the method identifies two clusters (green/purple) from the downsampled dataset. The completeness is high as all cells in each of the original clusters map to only one of the new clusters (orange to green, red to purple). However, homogeneity is low as the purple cluster contains both blue and red cells. In contrast, in the second scenario (Clustering 2), the method finds four clusters. The result is homogeneous as all the original orange cells are in their own new cluster (green) and all red cells are in another new cluster (purple). However, the blue cells are split into new blue and yellow clusters, so completeness is low. Alternatively, the method could discover three clusters (Clustering 3) but under a different configuration of labelling. While all orange cells map to the green cluster, the blue and red cells map to two new clusters (purple/yellow), which partition across the original blue and red clusters. Thus homogeneity and completeness scores are low. Only in the perfect scenario (Clustering 4) where the method perfectly reclassifies on the downsampled data, all metrics would have a value of 1.0.

We provide further simulation examples in Supplementary Sections H and I.

### Existing unsupervised clustering methods fail to reliably detect rare cell populations

**Figure 4.**
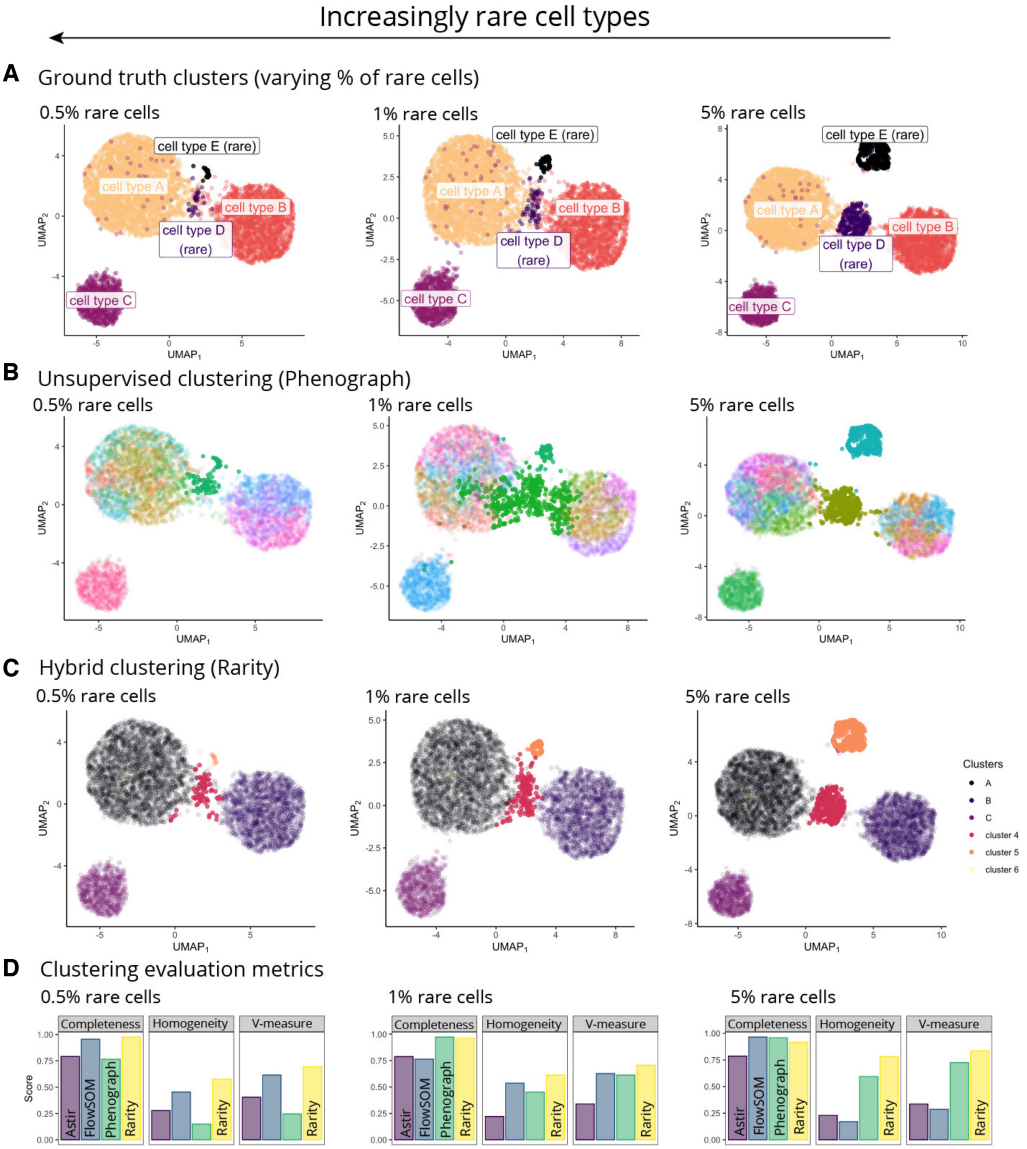
The less prevalent a cell type is, the more challenging it is to be reliably identified with unsupervised clustering methods. (A) Similarly to Fig. 1, ground truth data contain three common and two rare cell types. The three panels represent scenarios with varying extent of rarity: 0.5%, 1%, and 5% prevalence of rare cell types. (B) Unsupervised clustering with Phenograph (clusters shown in colour, highlighting the clusters which have the largest overlap with rare cell types) works well when the rare clusters are present at the 5% fraction; however, it has failed to identify one of the rare subpopulations at 1% presence and has only partially grouped the two rare cell types together at 0.5% prevalence. (C) Hybrid clustering with Rarity (clusters shown in colour) has correctly identified the two rare groups in all three scenarios. (D) To quantify clustering performance for (i) supervised (Astir), (ii) unsupervised (Phenograph), and (iii) hybrid (Rarity) methods, we display the conditional completeness, homogeneity, and V-measure scores (higher is better). An expanded version of this figure is given in Supplementary Section D.

We examined how effective existing commonly used unsupervised clustering methods (PhenoGraph and FlowSOM) were at detecting rare cell populations against the performance of Rarity. We generated artificial datasets consisting of five cell types (Fig. 4A) with three common (cell types A, B, C) and two rare (cell types D, E), where the latter were downsampled from an initial 5% of the cell population to 1% and then 0.5%. Using PhenoGraph (with the number of nearest neighbours set to the default value 30), we observed that common cell populations had the tendency to be fragmented into multiple clusters by the algorithm, while the ability to detect rare populations diminished as the population size decreased (Fig. 4B). With Rarity, common and rare cell populations were more reliably and consistently identified even with the decreasing rare cell population size (Fig. 4C). For comparison, we also assigned cell types with a supervised method, Astir, providing the marker information for all five cell types. Rarity (Fig. 4D) showed consistently superior performance compared to both PhenoGraph and Astir even though the latter is given the cell type profiles. While PhenoGraph maintains relatively high completeness, signifying that it tends to merge rare cell types into one cluster, homogeneity is low, as more than one cell type can be mapped to the same cluster. This illustrates the need for dual metrics to understand the complexities of interpreting clustering output, where the entire cluster structure may alter under different conditions. Details of further simulation experiments under different noise conditions are given in Supplementary Section B.

### Breast cancer IMC data

**Figure 5.**
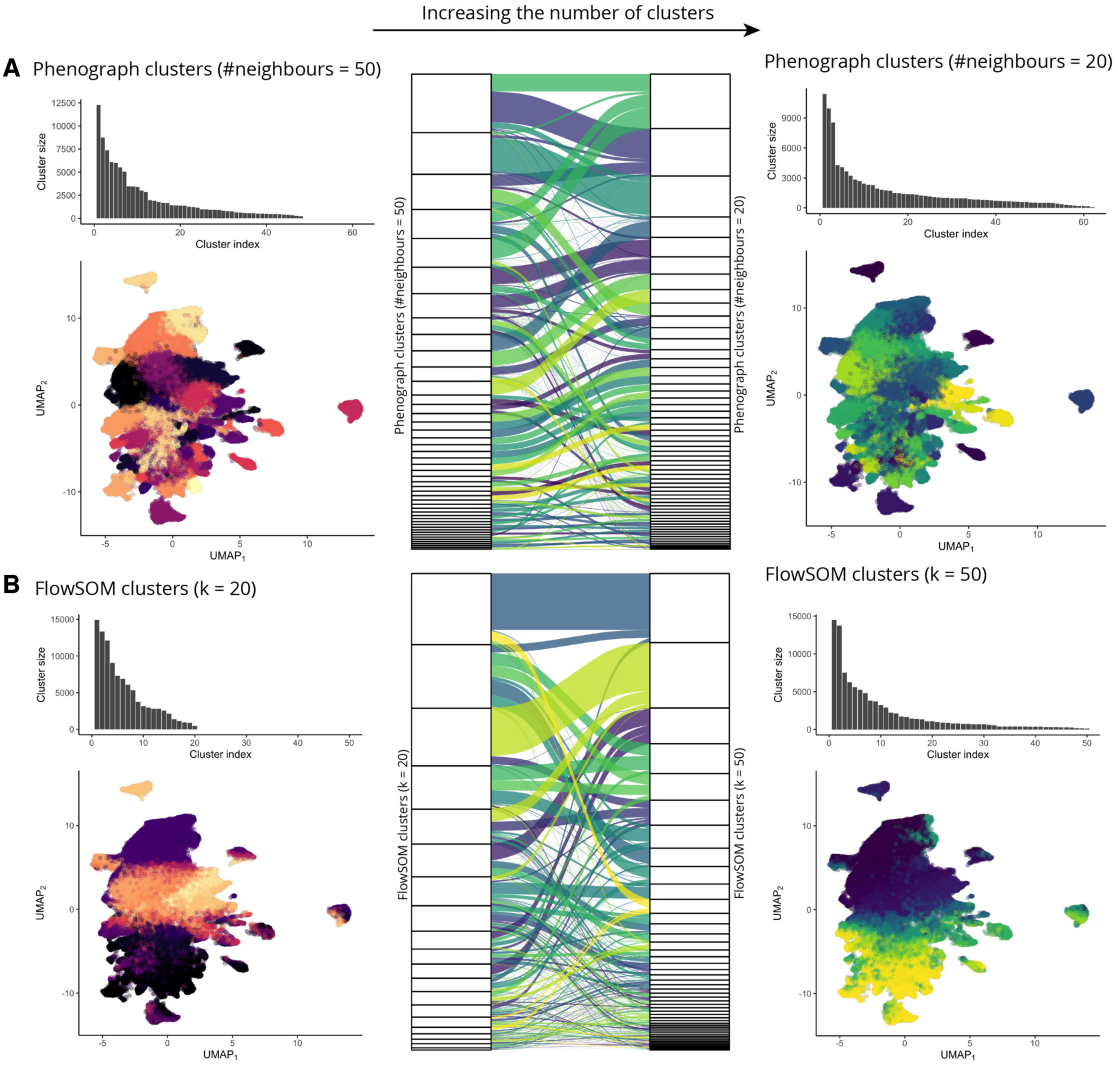
Unsupervised clustering algorithms have hyperparameters which let us either directly (FlowSOM) or indirectly (Phenograph) control the number of clusters, but clusters obtained under different hyperparameter configurations are not consistent to one another. (A) When we decrease the number of nearest neighbours in Phenograph from 50 (on the left) to 20 (on the right), we effectively increase the number of clusters; however, the mapping between the two sets of clusters (shown in the alluvial diagram in the middle) is highly complex and indicates inconsistency, as many clusters are both split and merged. (B) Similarly for FlowSOM, when we increase the number of clusters from 20 (on the left) to 50 (on the right), we observe both cluster splitting and merging.

We next considered a breast cancer IMC dataset (Jackson *et al.* 2020) and conducted an analysis using PhenoGraph and FlowSOM (Van Gassen *et al.* 2015) specifically looking at their ability to identify novel rare subpopulations before considering the utility of Rarity. Both PhenoGraph and FlowSOM possess algorithmic parameters, which can be modified to enable these methods to produce different numbers of output clusters—including potentially those corresponding to putative small subpopulations. Figure 5 shows how the number of clusters reported by PhenoGraph (Fig. 5A) and FlowSOM (Fig. 5B) varies with these parameters.

When the PhenoGraph hyperparameter corresponding to the number of neighbours is reduced from 50 to 20 (Fig. 5A), the number of clusters discovered increases from 48 to 62 and there is an increase in the number of clusters, which represent less than 1% of the total population from 24 to 35. In contrast, in FlowSOM we can explicitly control the number of clusters reported and we demonstrate this when increasing this number from 20 to 50 (Fig. 5B). This change led to an increase in the number of clusters, which represent less than 1% of the total population from 3 to 30. In general, hyperparameter adjustment alone does not allow these clustering approaches to become more sensitive to rare clusters in a readily interpretable way (see Supplementary Section C).

We next sought to understand how the underlying cluster structure alters as cluster number changes. Figure 5 illustrates how individual cell cluster assignments vary with output cluster number for FlowSOM and PhenoGraph. As cluster number changes, there are substantial cluster structure alterations with some clusters merging and splitting as more clusters are returned. For PhenoGraph, there was an average of 11 parents from the original clustering for each of the 62 clusters, while each of the 50 FlowSOM clusters had 7 parents. This indicates that for both methods, increases in the number of clusters did not increase sensitivity and the detection of small rare populations; instead, it led to fundamental changes in clustering structure.

**Figure 6.**
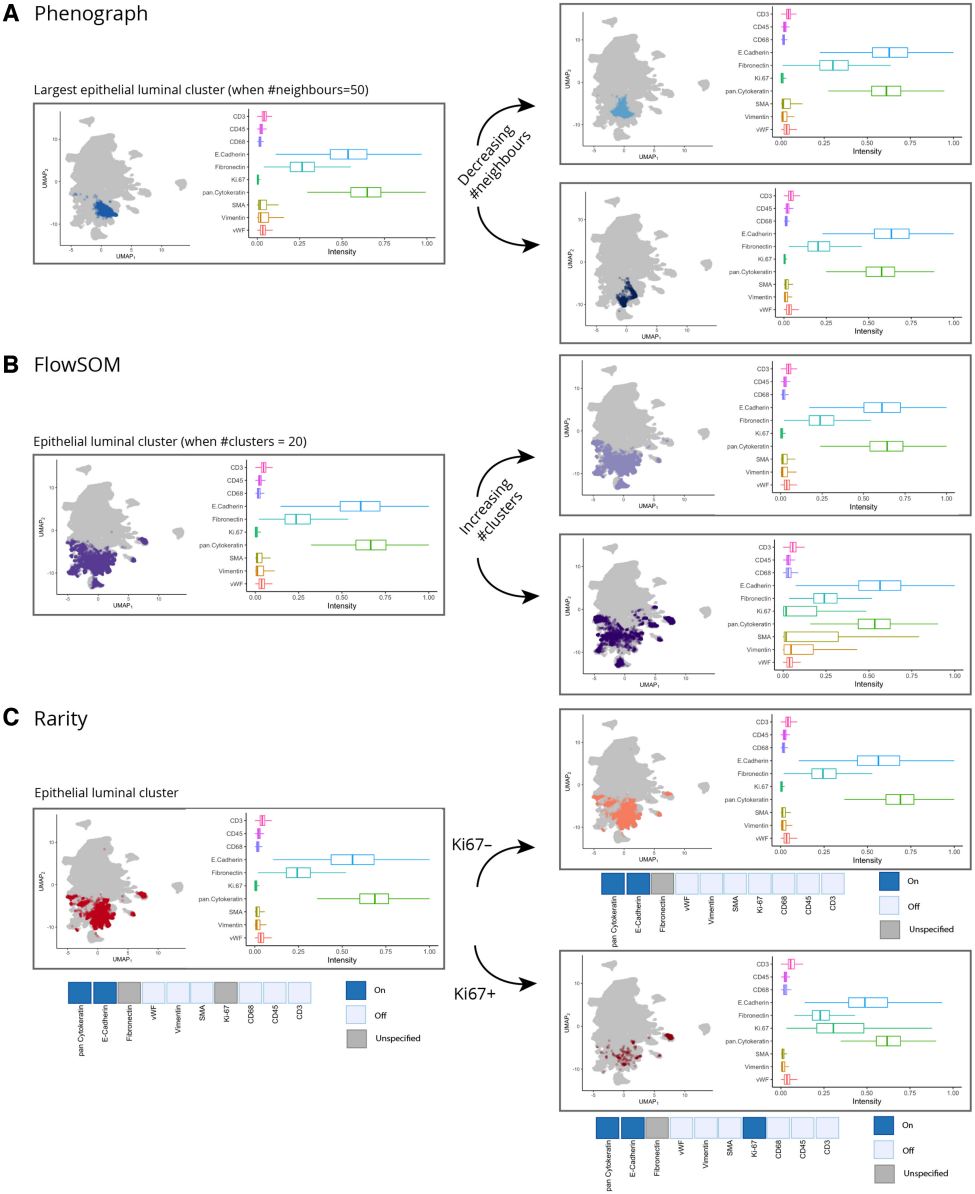
Increasing the number of clusters in unsupervised clustering methods does not typically help with identifying subclusters with distinct expression signatures. In all panels (A–C), we show the largest epithelial luminal cluster identified by (A) Phenograph, (B) FlowSOM, (C) Rarity, both on the UMAP plot and on the corresponding expression values boxplot. When increasing granularity (the clusters shown in the right column), the subpopulations identified by Phenograph are both extremely similar. For FlowSOM, a subpopulation expressing moderate levels of various markers (Vimentin, SMA, Ki67) emerges. In contrast, Rarity is the only method, where increase in granularity is directly interpretable in terms of expressing/not expressing a marker. The split illustrates how a Ki67+ subpopulation of epithelial luminal cells can be identified with Rarity.

When examining how particular clusters are split in terms of marker expression levels, Fig. 6A shows an illustrative example focusing on epithelial luminal cells, where PhenoGraph has generated two daughter clusters from a parent cluster. Each daughter cluster differs from the other only via a subtle change in the expression of two markers. For a similar group of epithelial luminal cells, FlowSOM partitions the cluster into two daughter clusters with entirely different expression signatures (Fig. 6B). These outputs are challenging to interpret as cluster structure and properties fundamentally alter as greater sensitivity is encouraged.

The hybrid approach of Rarity leads to a different approach to cluster interpretation. Since IMC profiles are mapped to latent binary expression vectors, we can select cells that match a particular expression pattern to determine clusters. Here, we were able to select a group of epithelial luminal cells by interrogating all cells that were mapped by Rarity to any latent binary vectors that express E-cadherin and pan-cytokeratin, but do not express e.g. CD45 or CD3 (Fig. 6C, full list of markers provided in Supplementary Table S5). We can then examine this set of cells and ask which cells among these do and which do not express Ki-67 by creating a more refined query for binary expression vectors, now additionally accounting for the presence and absence of Ki-67 expression.

**Figure 7.**
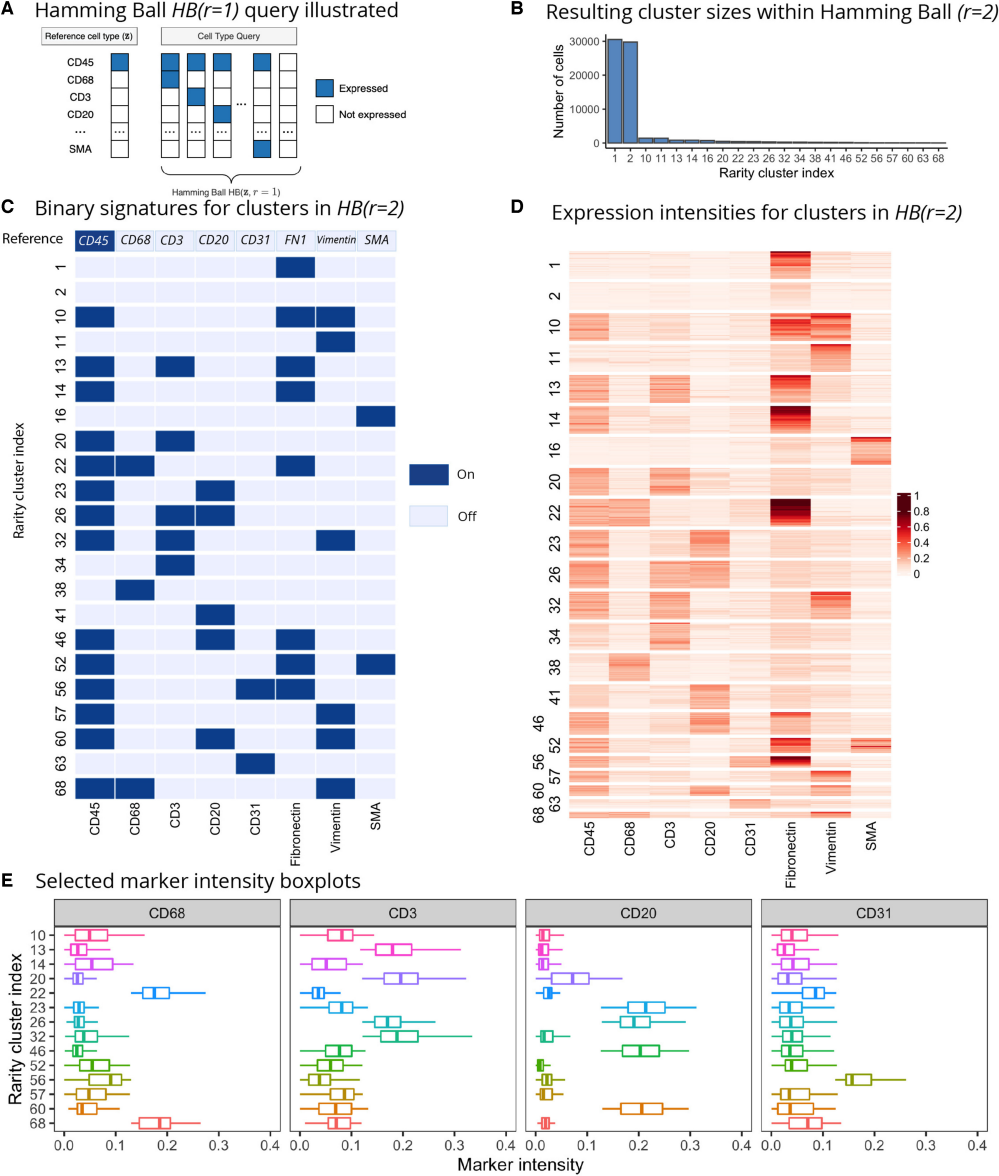
(A) Hamming ball query with radius r=1 illustrated: given a reference signature that expresses the CD45 marker, the query returns a set of binary signatures that differ from this reference at one (r=1) or two (r=2) markers. Panels (B–E) show the results for a Hamming ball query with the same reference but now r=2. (B) The number of cells in each cluster within the Hamming ball. (C) The binary signatures for all clusters within the Hamming ball, and (D) the corresponding observed intensities heatmap (for visual aid, we display a subset of the two largest clusters on the heatmap). (E) Selected marker intensity boxplots (CD68, CD3, CD20, and CD31) for the identified clusters (shown for all clusters that express CD45) are in concordance with the binary profiles shown in (C) and highlight how every cluster has a unique interpretation in terms of differential expression signatures.

With Rarity, each cell is mapped onto a cluster with a clear latent binary expression pattern, where each cluster (by definition) must at least differ by the expression of one marker (Fig. 6C, Fig. 7) and leads to a natural hierarchy of clustering assignments.

Next we demonstrate further how Rarity can help us identify a more granular clustering, which can give insights into finding putative rare cell subpopulations. One way to explore the subpopulations identified by Rarity is via what we call the ‘Hamming ball query’ (Fig. 7A), where we search for binary expression signatures that deviate from the signature of a reference cell type no more than a given number of markers (i.e. their Hamming distance from the reference signature does not exceed a given radius). For example, Fig. 7A illustrates a query where the reference signature corresponds to cells that express the CD45 marker. This query would let us identify various immune cells;e.g the Hamming ball with radius r=1 would contain macrophages, i.e. a signature where both CD45 and CD68 markers are expressed.

This functionality shows how Rarity can complement the cell type identification with a supervised method such as Astir (Geuenich *et al.* 2021) where all the cell types of interest have to be prespecified a priori. In the case of Astir, there is an additional ‘Other’ cluster that will consist of a mix of unrecognized cell types. Figure 7 shows how Rarity has found substructure within this ‘Other’ group of cells. Specifically, we aim to distinguish between different classes of stromal cells: immune cells, endothelial cells, smooth muscle, and fibroblasts. We have conducted analysis starting from the reference signature with CD45 as the main immune cell marker (binary expression signature shown in the top row of panel of Fig. 7C), and considering all cell type signatures within the Hamming ball with radius r=2. For example, the first (and largest) cluster does not express the CD45 marker but does express fibronectin, suggesting that it corresponds to a set of cells from the connective tissue. Clusters that co-express both CD45 and fibronectin/vimentin (i.e. clusters 10, 11, 13, 14, 22, 32, etc.) are likely to be immune cells that are located within the stroma, e.g. cluster number 13 would correspond to such T cells. Zooming in to clusters that express CD68 (i.e. clusters 22, 38, 68) helps us identify macrophages. Rarity has also identified a group of vascular cells (clusters 56 and 63 which express the CD31 marker). The heatmap (Fig. 7D) and boxplots (Fig. 7E) confirm that indeed the inferred binary signatures are indicative of the actual intensity levels, thus aiding interpretability.

### Downsampling experiments highlight how most clustering methods are inconsistent

**Figure 8.**
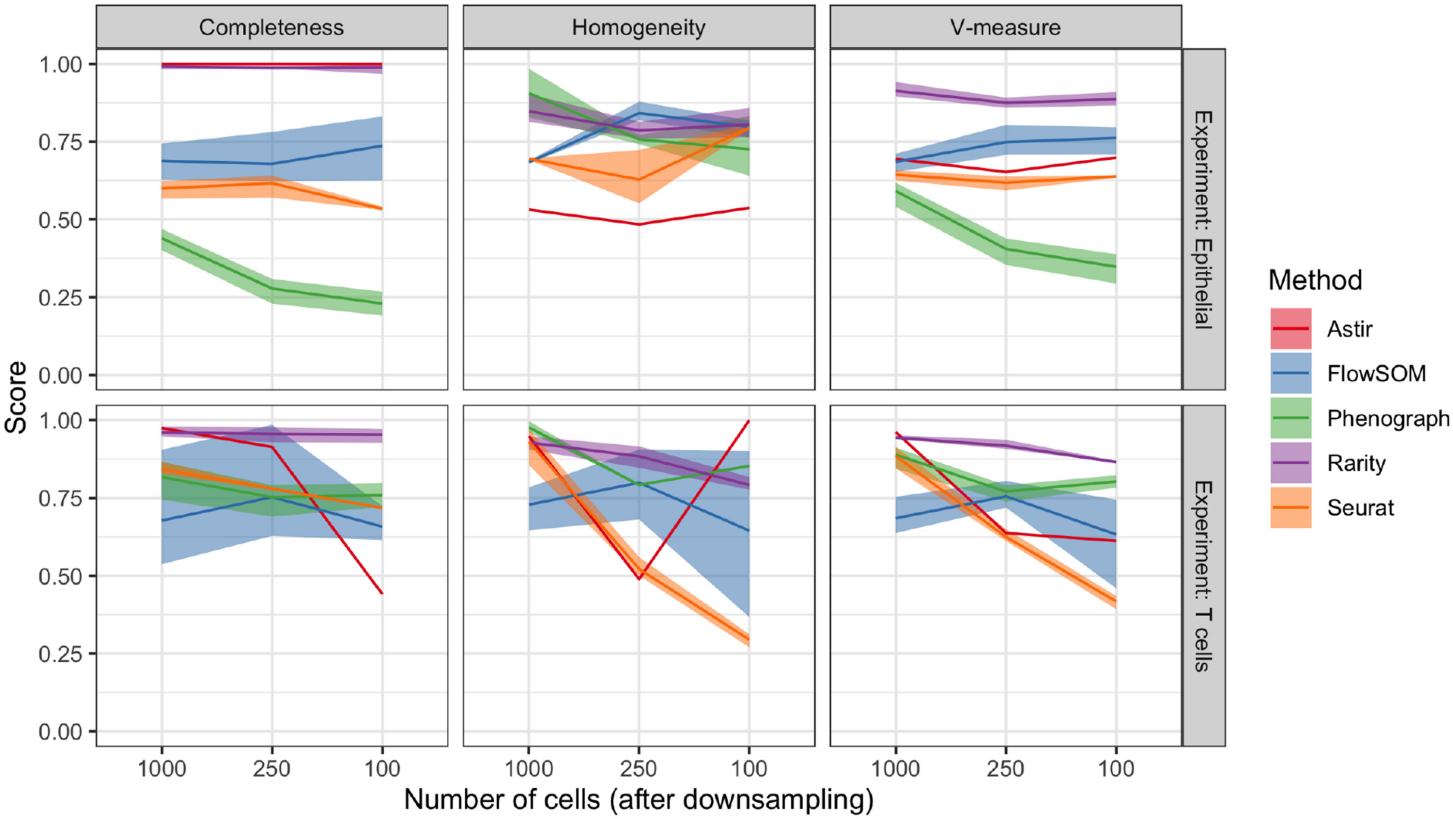
Clustering scores (conditional completeness, homogeneity, and V-measure) when downsampling the largest epithelial (top row) or T cell (bottom row) cluster identified by the respective methods (Astir, FlowSOM, Phenograph, Rarity, Seurat), varying the number of cells retained after downsampling from 1000 to 250 to 100 (x-axis). The scores quantity the goodness of the clustering with respect to the respective methods run on the full dataset, thus quantifying self-consistency.

We next performed a downsampling experiment using the same breast cancer IMC data to further characterize the rare cell detection capabilities of each method. We applied FlowSOM, PhenoGraph, Louvain clustering implemented within Seurat v3 (Stuart *et al.* 2019), as well as the supervised method Astir together with Rarity to the data. Each method identified different clustering configurations and hence different cell type numbers. However, from the output of each method, we identified the clusters most likely to correspond to epithelial or T cells (using the expression of E-cadherin and pan-cytokeratin for epithelial, and CD45, CD3 for T cells). For Astir, these markers were used to predefine the cell signature to be identified as input. We then created downsampled datasets: first, downsampling epithelial cells in one experiment and T cells in the next. These cell types were reduced from the original numbers to 1000, 250, and 100 cells, respectively (Fig. 8). The clustering methods were then reapplied to the downsampled data to determine if the clustering labels from the full dataset are recapitulated. Thus, these experiments do not rely on any ‘ground truth’ cell type labels, and they simply indicate how consistent every method on its own is.

Rarity showed stable and superior clustering performance to that of other unsupervised approaches (Fig. 8). We observed that the sensitivity of unsupervised methods did not simply decline with decreasing numbers of cells, but that the performance characteristics were more complex and there was a dependency on cell type. This is due to the fact that clustering output from the same method on original and downsampled datasets often exhibited significantly different clustering output (similarly to the previous synthetic data experiments), while in contrast Rarity’s design makes it less susceptible to this. Intriguingly, while Astir is a supervised method and is given target gene signatures as input, its performance was also not always stable across downsampled datasets. This is due to the fact that certain model parameters are inferred dynamically, so its performance is also partially dataset dependent in this case with T cells.

### Rarity identifies CD4-negative and CD8-negative T cells in colorectal cancer IMC data

We next examined the utility of Rarity for the discovery of gamma-delta T cells in immunogenomic profiles of normal colon mucosa in patients with multiple or single and familial or sporadic colorectal cancer. IMC data were generated for sixteen samples of non-cancerous colon mucosa obtained from six individuals who underwent surgical resection of colorectal cancers (see section ‘Methods’ for full experimental details). The imaging data were originally published as part of (Bortolomeazzi *et al.* 2022), but now we have additionally made available the processed single-cell dataset (Märtens *et al.* 2022). Gamma-delta T cells were expected to constitute less than 10% of T cells in human colon mucosa (Viney *et al.* 1990), and their characteristic feature is that they are CD3-positive but both CD4-negative and CD8-negative. However, when using standard supervised and unsupervised methods applied to this dataset, no such clusters are identified. This is not surprising in the light of our simulation study in Fig. 4, even if those cell types were present. Therefore, we wanted to see if the increased sensitivity in Rarity will identify any potential candidates for such CD4– and CD8– double-negative T cells.

**Figure 9.**
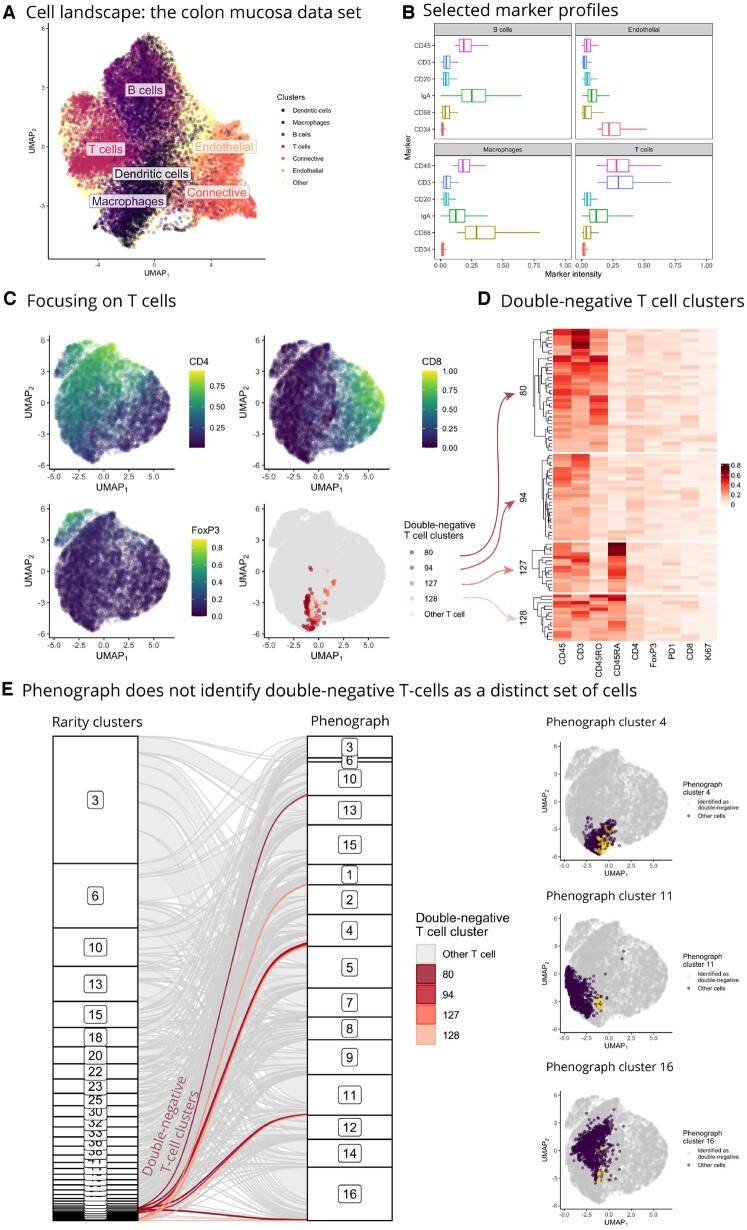
(A, B) Cell landscape UMAP plot, where we have highlighted cells identified as B cells, T cells, macrophages, dendritic, endothelial, and connective tissue cells, together with the corresponding marker intensity boxplots. Next, panels (C–E) focus on analysing T cells only. (C) UMAP plots of T cells showing expression of CD4, CD8, and FoxP3, and highlighting the location of identified CD4– CD8– T cells (i.e. gamma-delta T cell clusters) by Rarity. (D) Marker expression levels for the identified CD4– CD8– T cell subgroups (i.e. Rarity clusters 80, 94, 127, 128) show combinatorial co-expression of CD45RA and CD45RO. (E) These double-negative T cell clusters would not have been identified by unsupervised clustering with Phenograph. In fact, Phenograph has placed these cells into various larger clusters (e.g. clusters number 4, 11, 16, etc.) as shown in the alluvial diagram (highlighting the four Rarity clusters) and UMAP plots for selected Phenograph clusters (highlighting the double-negative cells in yellow).

After preprocessing steps (see section ‘Methods’ for details), our colon mucosa dataset contains a total of 40 364 cells. We used Rarity to classify these cells into B cells, T cells, macrophages, dendritic cells, endothelial, connective tissue cells, and other cells, as illustrated in Fig. 9A, using markers listed in Supplementary Table S6. For selected markers and cell types, Fig. 9B shows the corresponding marker intensity boxplots.

Next, we turned to the analysis of T cells. That is, the following analysis is restricted to the cells identified by Rarity as CD45-positive and CD3-positive. Figure 9C displays the UMAP when refitted on T cells only. We can see that CD4 intensity increases along the y-axis, CD8 intensity increases along the x-axis, and the blob in the top left corner corresponds to Regulatory T cells expressing FoxP3. The fourth subpanel highlights the set of T cells that were identified by Rarity as CD4− and CD8− cells that could potentially be gamma-delta T cells. In fact, Rarity identified four such clusters (clusters number 80, 96, 127, and 128). Heatmap in Fig. 7D confirms that these clusters have indeed low intensity levels of CD4 and CD8, but it additionally provides insight into why there are four clusters instead of a single one—these clusters are stratified by CD45RO and CD45RA marker intensities, indicating memory and naive T cells, respectively. In total, the putative gamma-delta T cell clusters lacking CD4 and CD8 comprise 92 cells (which corresponds to 0.2% of all cells and 1.0% of all T cells).

**Figure 10.**
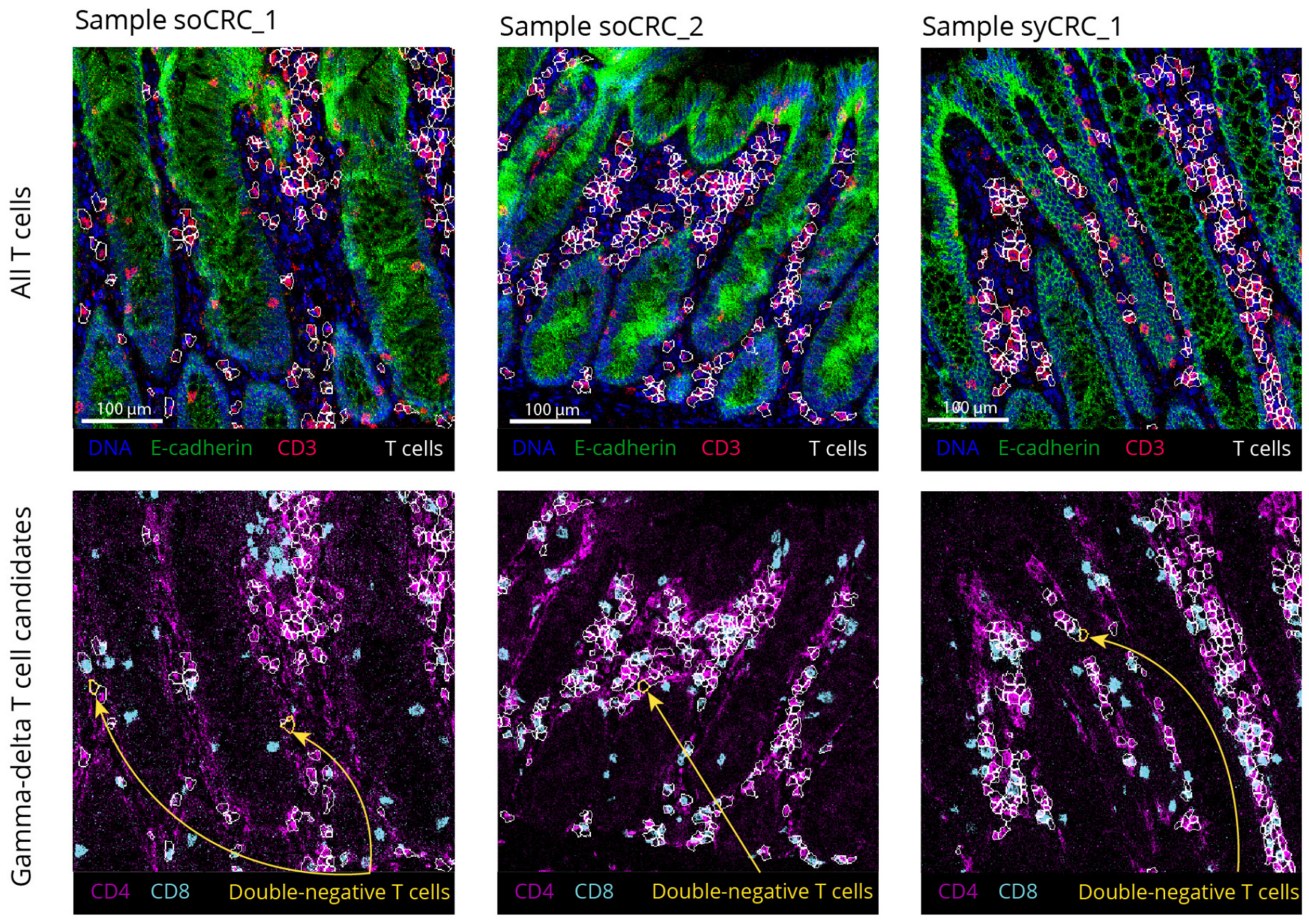
Examples of double-negative T cells identified by Rarity displayed on the original IMC images. For a zoom-in of three samples (soCRC_1_, soCRC_2_, and syCRC_1_), the top row shows DNA (blue), E-cadherin (green), and CD3 (red) marker intensities, and highlights T cell outlines (in white). The bottom row shows CD4 (magenta) and CD8 (cyan) marker intensities, and highlights double-negative T cells (yellow outlines, also indicated with arrows) among all T cells (in white).

We re-emphasize that these clusters were not identified as a distinct set of cells on the UMAP visualization, a phenomenon that we already observed earlier in Figs 1 and 4. Furthermore, this set of cells would not have been discovered by an unsupervised method like Phenograph. Figure 10E illustrates how cells in these CD4– and CD8– T cell clusters are spread across multiple larger Phenograph clusters—a behaviour consistent with our earlier findings.

We further explored the candidate gamma-delta T cell by mapping these to their spatial locations and samples of origin. We found that the 92 identified cells were uniformly spread across biological samples with on average six cells per sample. This confirms that the cells were not due to an experimental artefact specific to a subset of samples. Figure 10 displays the spatial location of the identified gamma-delta T cells in three samples. These were located in the subepithelial areas of diffuse connective tissue of the lamina propria (Hayday and Gibbons 2008, Kurd and Robey 2014) consistent with the known distribution of such cells and their association with intraepithelial sites.

## Discussion

Our motivation for the development of Rarity stemmed from the need to identify rare cell types from single-cell data. In this work, we have systematically demonstrated how commonly used clustering methods fail to discover clusters that are rare but yet distinct—our downsampling experiments showed how the task of cell type identification becomes increasingly difficult the less prevalent the cell type becomes. Our simulation experiments also highlight a lack of self-consistency in current unsupervised clustering algorithms.

Given the plethora of literature on clustering methods for cell type detection, rare cell type identification has received relatively much less attention. For example, both (Cron *et al.* 2013, Naim *et al.* 2014) have used a variation of the Gaussian mixture model, with different strategies for choosing the number of clusters. An increased number of clusters will lead to higher sensitivity towards rare subpopulations; however, these methods have not been inherently designed to recognize rare groups specifically. As we have demonstrated in the paper, simply increasing the number of clusters does not generally lead to the desired outcome. Instead, increased sensitivity typically leads to breaking existing large clusters down into smaller ones with miniscule differences in their respective expression signatures. Our findings are aligned with those highlighted by (Weber and Robinson 2016) who also found that clustering results can be highly variable and, e.g. sensitive to bootstrap resampling.

With Rarity, we have set out a bespoke approach for delineating these cellular groups. This was based on the idea of transforming the single-cell data into high/low binary expression patterns, which induces an implicit clustering of the cells. We demonstrated how this approach led to a robust system for identifying rare cell types, and the use of binary expression patterns provides a certain form of guarantee that the clustering output is highly interpretable (i.e. that each cluster must differ from the others by at least one molecular feature). We recognize the limitation of this assumption is that cell types that differ from others only via changes in absolute levels of expression would not be identified by Rarity. The limitations are explored in Supplementary Section F.

## Conclusion

Rarity has complementary utility to existing cell type discovery methods. Rarity makes stronger assumptions about the expression patterns of distinct cell types, sacrificing sensitivity to subtle differential expression patterns to reduce the number of false positive cluster findings in order to maximize the chance of finding rare, but distinctive, cell types. We believe this construction makes Rarity particularly useful in an interactive setting in which analysts are able to manually navigate the clustering findings through Hamming ball queries as illustrated in the examples.

Rarity has been implicitly designed for use with targeted molecular profiling technologies with data dimensions on the order of 10–40 features. We believe that in such settings, the measured molecular features will have been chosen to be cell type markers, which makes the latent binary expression assumptions in Rarity more applicable. While, in principle, our methodological framework is general and could be extended to analyse single-cell RNA sequencing (scRNA-seq) data, considerably more care is required with the definition of ‘rare’ cell types in high-dimensional settings. For instance, any small similar group of cells which differs from any other cell type by just a single gene could—in principle—be a candidate rare cell type. Given that a whole transcriptome analysis will yield 10 000s of genes, the possibility of large numbers of false clusters is substantial.

While a number of approaches have been developed with rare cell type identification capability for scRNAseq, including RaceID3 (Grün *et al.* 2016), GiniClust2 (Tsoucas and Yuan 2018), GapClust (Fa *et al.* 2021), CellSIUS (Wegmann *et al.* 2019), FiRE (Jindal *et al.* 2018), and scAIDE (Xie *et al.* 2020), like in single-cell cytometry, these approaches are predominantly for unsupervised discovery of larger cell populations and therefore will be sensitive to many of the issues we have discussed. Further research is required to devise a general framework for defining clustering criteria or (dis)similarity metrics that target specific cell phenotypic properties. We leave this as an open challenge to the community.

## Methods

### Conditional metrics for homogeneity and completeness

Suppose we have *C* true cell types and *K* inferred clusters. Let *A* be the contingency table where entry $a_{ck}$ denotes the number of cells corresponding to cell type *c* in cluster *k*, where the total number of cells is *N*.

Analogously to Rosenberg and Hirschberg (2007), we now define *conditional* homogeneity, completeness, and V-measure metrics which would let us ‘zoom in’ to the rare clusters of interest as opposed to averaging across all clusters.

We first define the conditional entropies as follows:

$$\begin{array}{c} H(K|C=c)=-\sum_{k}\frac{a_{ck}}{\sum_{c}a_{ck}}\;\text{log}\;\frac{a_{ck}}{\sum_{c}a_{ck}},\\ H(K|K=k)=-\sum_{c}\frac{a_{ck}}{\sum_{k}a_{ck}}\;\text{log}\;\frac{a_{ck}}{\sum_{k}a_{ck}}, \end{array}$$

as well as the marginal entropies

$$\begin{array}{c} H(C)=-\sum_{c}\frac{\sum_{k}a_{ck}}{N}\;\text{log}\;\frac{\sum_{k}a_{ck}}{N},\\ H(K)=-\sum_{k}\frac{\sum_{c}a_{ck}}{N}\;\text{log}\;\frac{\sum_{c}a_{ck}}{N}, \end{array}$$

We are now interested in defining *conditional* metrics to quantify the quality of the clustering w.r.t. the (potentially rare) cell type. We achieve this by defining the conditional completeness

$$c-\text{completeness}:=1-\frac{H(K|C=c)}{\max\;H(K)}$$

and the conditional homogeneity as

$$c-\text{homogeneity}:=1-\frac{H(C|K=k*)}{\max\;H(C)}$$

where $k*=\arg\;\max_{k}\;a_{ck}$ and the latter is conditional on the most likely cluster k* for cell type *c*. Finally, we define the conditional V-measure as the harmonic mean of the respective conditional completeness and homogeneity scores.

Supplementary Section G provides further information about the importance of these metrics as supposed to classification metrics.

### Rarity model specification

Rarity has been designed to trade off sensitivity with respect to small cell populations and interpretability. To achieve this goal, Rarity relies on a modelling assumption that every gene is either expressed or not, and these binary states are captured via binary latent variables. Rarity implements inference for these underlying binary states. Clustering is induced by these binary signatures: cells with identical binary signatures are grouped together.

We associate every observed gene expression vector $x_{i}\in R^{P}$ with an underlying latent variable $z_{i}\in {\left\{0,1\right\}}^{P}$ with binary entries $z_{ig}∼\text{Bernoulli}(\cdots )$, where $z_{ig}=1$ corresponds to gene *g* in cell *i* being expressed and $z_{ig}=0$ to being not expressed. We specify the likelihood conditional on the latent variable as follows:

$$p(x_{ig}|z_{ig})=(1-z_{ig})N(\mu_{1},\sigma_{1}^{2})+z_{ig}N(\mu_{2},\sigma_{2}^{2}),$$

where the first component $N(\mu_{1},\sigma_{1}^{2})$ represents the distribution of markers that are ‘not expressed’ and the second one $N(\mu_{2},\sigma_{2}^{2})$ represents those that are ‘expressed’.

To make inference for this binary latent variable model tractable and scalable, we employ continuous relaxations for the binary variables in the Variational Autoencoder framework. That is, we reformulate the model as follows:

$$\begin{array}{c} z_{ig}∼\text{RelaxedBernoulli}(p=0.5),\\ x_{ig}|z_{ig}∼(1-z_{ig})N(\mu_{1},\sigma_{1}^{2})+z_{ig}N(\mu_{2},\sigma_{2}^{2}) \end{array},$$

and we perform amortized variational inference with the approximate posterior $q(z_{i})=\text{RelaxedBernoulli}(f_{\phi }(x_{i}))$, where $f_{\phi }$ is an encoder neural network with shared variational parameters ϕ.

### Synthetic data generation

For the synthetic IMC data example, we used the following simulation scheme. We first generated the underlying binary vectors

$$\begin{array}{c} z_{\text{cell type}\;A}=(1,1,1,1,0,0,0),\\ z_{\text{cell type}\;B}=(1,1,1,0,1,0,0),\\ z_{\text{cell type}\;C}=(1,1,1,1,0,1,0),\\ z_{\text{cell type}\;D}=(1,1,1,0,0,0,0),\\ z_{\text{cell type}\;E}=(1,1,0,0,0,0,0), \end{array}$$

where ‘1’ indicates that a marker is ‘on’ and ‘0’ that it is ‘off’. Conditional on these binary signatures, we then generated the observations as follows:

$$x_{i}|z_{c_{i}=k}∼N(0.5z_{k}+0.05(1-z_{k}),0.18z_{k}+0.03(1-z_{k}))$$

for cell type k∈{A,B,C,D,E}, where the number of cells for each cell type is, respectively, 4000, 3000, 1000, 60, and 40.

### Colon mucosa image mass cytometry

#### Sample description

Sixteen Formalin-Fixed Paraffin-Embedded (FFPE) blocks of non-cancerous colon mucosa (Supplementary Table S3) were obtained from six individuals who underwent surgical resection of colorectal cancers, and subsequently reviewed by an expert pathologist. All patients provided written informed consent in accordance with approved institutional guidelines (University College London Hospital, REC Reference: 20/YH/0088; Istituto Clinico Humanitas, REC Reference: ICH-25–09).

The experimental and computational processing of the colon mucosa IMC data was previously described in (Bortolomeazzi *et al.* 2022). Here, we provide a brief summary of the staining and image analysis steps:

#### Staining and IMC ablation

A microtome was employed to cut one 4-μm-thick section from each of the FFPE blocks from all samples. These sections were stained with a panel of 26 antibodies, targeting the main cell populations of the colon mucosa including immune, stromal, and epithelial cells (Supplementary Table S4). The optimal dilution for each antibody was selected by a mucosal immunologist after reviewing the images generated from the ablation and staining of FFPE appendix sections at different concentrations (Supplementary Table S3). Before staining, slides were incubated for 1 h at 60°C, dewaxed, rehydrated, and then underwent antigen retrieval. This was performed in a pressure cooker with Antigen Retrieval Reagent-Basic (R & D Systems). Then slides were blocked by incubating them in a 10% BSA (Sigma), 0.1% Tween (Sigma), and 2% Kiovig (Shire Pharmaceuticals) Superblock Blocking Buffer (Thermo Fisher) blocking solution at room temperature for 2 h. The selected concentration of each antibody was added to a primary antibody mix in blocking solution and incubated overnight at 4°C. Then, the slides were washed twice in PBS and PBS-0.1% Tween and incubated for 30 min with the DNA intercalator Cell-ID™Intercalator-Ir (Fluidigm) (containing the two iridium isotopes 191Ir and 193Ir) 1.25 mM in a PBS solution. Subsequently, the slides were washed once in PBS and once in MilliQ water and air-dried.

The Hyperion Imaging System (Fluidigm) imaging module was used to obtain a light-contrast high-resolution image of approximately 4 mm^2^ of each stained slide. These images were used to select the region of interest (ROI) in each slide. One square millimetre ROIs were selected to contain the full thickness of the colon mucosa in a longitudinal orientation and ablated at a 1 μm/pixel resolution and 200 Hz frequency.

#### IMC Image processing and data preprocessing

The ablation generated raw .txt and .mcd files, from which 28 images from 26 antibodies (Supplementary Table S4) and two DNA intercalators were extracted with imctools (https://github.com/BodenmillerGroup/imctools). Pixel intensities for each channel were normalized to the 99th percentile in all samples with custom R scripts, and background pixels were removed by thresholding with CellProfiler 2 (Kamentsky *et al.* 2011). A mask for the lamina propria was manually drawn for each sample using the vimentin channel as a guide and reviewed by a mucosal immunologist.

Cells segmentation was performed first by identifying nuclei on a thresholded image derived from the multiplication of the two DNA channels. The nuclei were then used as seeds for propagation on a membrane mask derived from the sum of the E-cadherin, CD45, CD3, CD4, CD8, CD45RO, CD27, CD68, CD34, and SMA channels. The resulting cells were then filtered according to their overlap with the lamina propria mask. Only cells overlapping the mask by more than 50% of their area were retained, resulting in a total of 40 364 cells. Finally, the mean pixel intensity of each marker was measured in each cell.

## Supplementary Material

btad750_Supplementary_Data

## Data Availability

The datasets analysed during the current study are available from the Zenodo repository: (1) Breast Cancer (https://zenodo.org/record/4607374#.YgbYffXP2Lo) and (2) Colon Cancer (https://zenodo.org/record/5545882#.Yh853RPP04g).
