# Supplementary material for: Rarity: discovering rare cell populations from single-cell imaging data
Source: Bioinformatics. 2023 Dec 13;39(12):btad750. doi: 10.1093/bioinformatics/btad750 (PMC10751233; doi:10.1093/bioinformatics/btad750)
Supplement: btad750_Supplementary_Data [file btad750_supplementary_data.zip › supp.pdf]

# Supplementary Information
# Rarity: Discovering rare cell populations from single-cell imaging data

Kaspar Märtens[1], Michele Bortolomeazzi[2,3], Lucia Montorsi[2,3], Jo Spencer[4], Francesca Ciccarelli[2,3], and Christopher Yau[1,5,6]

[1] *The Alan Turing Institute, London, UK*
[2] *Cancer Systems Biology Laboratory, Francis Crick Institute, London, UK*
[3] *School of Cancer and Pharmaceutical Sciences, King's College London, London, United Kingdom*
[4] *School of Immunology and Microbial Sciences, King's College London, London, United Kingdom*
[5] *Health Data Research UK, London, UK*

## Contents

**A UMAP and t-SNE visualisations are not robust for identifying clusters** **2**

**B Performance under varying noise distributions** **2**

**C Hyperparameter tuning of existing clustering methods does not improve outcomes** **3**

**D Expanded version of Figure 4** **6**

**E Meta-clustering does not improve rare cluster identification** **6**

**F Sensitivity to cell types that differ by non-binary expression changes** **7**

**G Classification metrics are not suitable for performance benchmarking** **10**

**H Synthetic example: marker genes** **12**

**I Additional scenario: a rare cell type with many marker genes** **12**

# A UMAP and t-SNE visualisations are not robust for identifying clusters

In this illustration, we demonstrate why the use of UMAP (or t-SNE) visualisations for rare cluster identification purposes can be problematic. This is because UMAP visualisation will depend on both the random seed as well as hyperparameter choice. Here we consider values for parameters $\texttt{n\_neighbours} \in \{5, 15, 30\}$ and $\texttt{min\_dist} \in \{0.1, 0.3\}$ well within the ranges recommended by the original authors. We demonstrate this subtle instability by showing three different UMAP plots in Supplementary Figure 1, all visualising our synthetic dataset. While the three large clusters remain equally recognisable across all three plots, the shape and number of the small clusters containing cell types D and E are not consistent, as highlighted in Supplementary Figure 1.

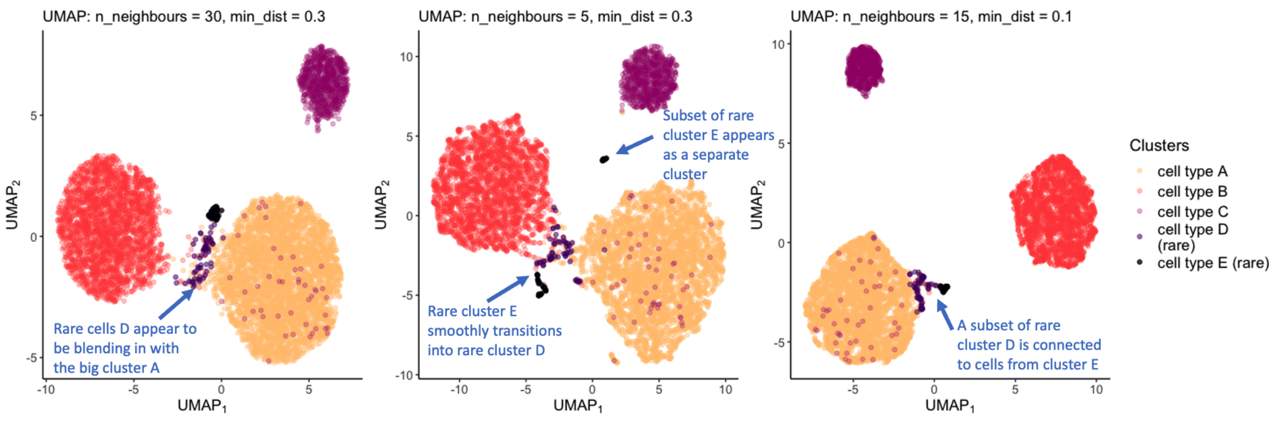


**Supplementary Figure 1:** The UMAP visualisation cannot be reliably used to detect the number of clusters, demonstrated across three hyperparameter configurations. While the three large clusters remain recognisable across all three plots, the shape and number of small clusters is not consistent.

To further demonstrate these effects on real data, we show the same three hyperparameter configurations on the double-negative T cell example in Supplementary Figure 2. While our previously identified double-negative T cells seem to be broadly covering the same area in UMAP, there are substantial changes to the local structure. We have quantified this by comparing the fraction of 10 nearest neigbour cells that remain consistent across the three UMAP plots, calculated for each of the double-negative T cells. For example, there are 25 (out of 92 total) double-negative cells for which *all* of the 10 nearest neighbours change between the 1st and the 2nd UMAP configuration. In light of these sources of variability, we did not consider manual inspection as a stable benchmark-able standard for identifying rare cell populations.

# B Performance under varying noise distributions

We conducted a simulation experiment where we quantify the amount of false positive discoveries under varying noise levels. Specifically, we considered Gaussian distributions $\mathcal{N}(\mu, \sigma^2)$ with fixed $\mu = 0.55$ and increasing $\sigma$ values $\sigma \in \{0.1, 0.15, 0.2\}$ as well as a log-normal distribution, as illustrated in Supplementary Figure 3(A). For all four distributions, we consider two scenarios: (B) “all markers” and (C) a “single marker” case. In (B), the expression intensities for all markers are drawn independently from one of the four specified distributions, whereas in (C), only a selected marker is drawn from the respective distributions[1]. The ground truth corresponds to a single cluster where all markers should be “on”. However, for increasing noise levels, there may be additional cluster configurations found.

As seen from panels Supplementary Figure 3(B, C), for increasingly large noise levels Rarity starts to identify false positive clusters (for ideal performance, all cells should be clustered into a single

[1] The rest are drawn from a low-variance base distribution, corresponding to scenario 1

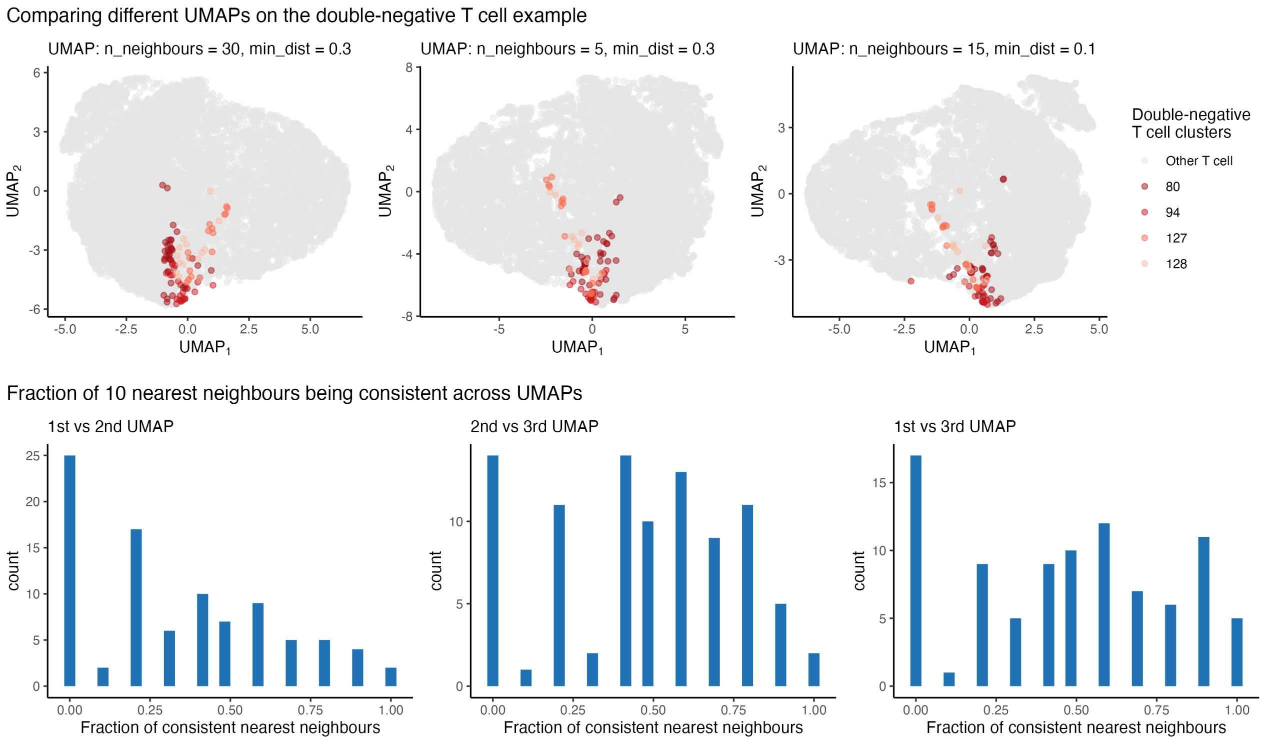


**Supplementary Figure 2:** While the overall *shape* of the UMAP point cloud remains similar across small changes in UMAP hyperparameters, the *local structure* substantially changes. We quantify this by comparing changes in the 10 nearest neighbours to all double-negative T cells. The bottom panels show the fraction of nearest neighbours that remain the same across the three UMAP plots. For example, there are 25 (out of 92 total) double-negative cells for which *all* of the 10 nearest neighbours change between the 1st and the 2nd UMAP configuration.

cluster where all markers are "on"). In the "all markers" scenario we observe a larger number of false positive rare cluster findings[2], in contrast to a "single marker" scenario. This is expected, as in the former case there can be different combinations of markers that exhibit larger deviations. For example, in the $\sigma = 0.2$ scenario in panel (B), 92.1% of cells (a total of 921 cells out of 1000) are correctly identified and 7.9% cells belong to false positive clusters. In the log-normal scenario in (B), 97.3% of cells (973 cells out of 1000) were correctly identified. For the single marker case, the fraction of correctly specified cells across the four scenarios is 100%, 100%, 99.2% and 99.7% respectively.

While "all markers" and "single marker" are two extreme scenarios, we expect realistic scenarios to lie in-between the two.

These examples demonstrate that for high noise levels, it is possible that Rarity identifies clusters when the data generative process did not involve any structure. While it is important to keep this implication in mind when applying Rarity in practice, this property is not unique to our method – in fact, we would expect it to affect most clustering methods.

## C Hyperparameter tuning of existing clustering methods does not improve outcomes

In the scenarios where ground truth cell type labels are available, one can try to find optimal hyperparameters for PhenoGraph and FlowSOM to maximise the desired metric, such as the (conditional) V-measure. In this experiment, we vary the $k$ parameter for the synthetic example (from Figure 4), with varying proportions (0.5%, 1%, 5%) of rare cells, as shown in Supplementary Figure 4.

[2] Note that the $y$-axis is shown on log-scale

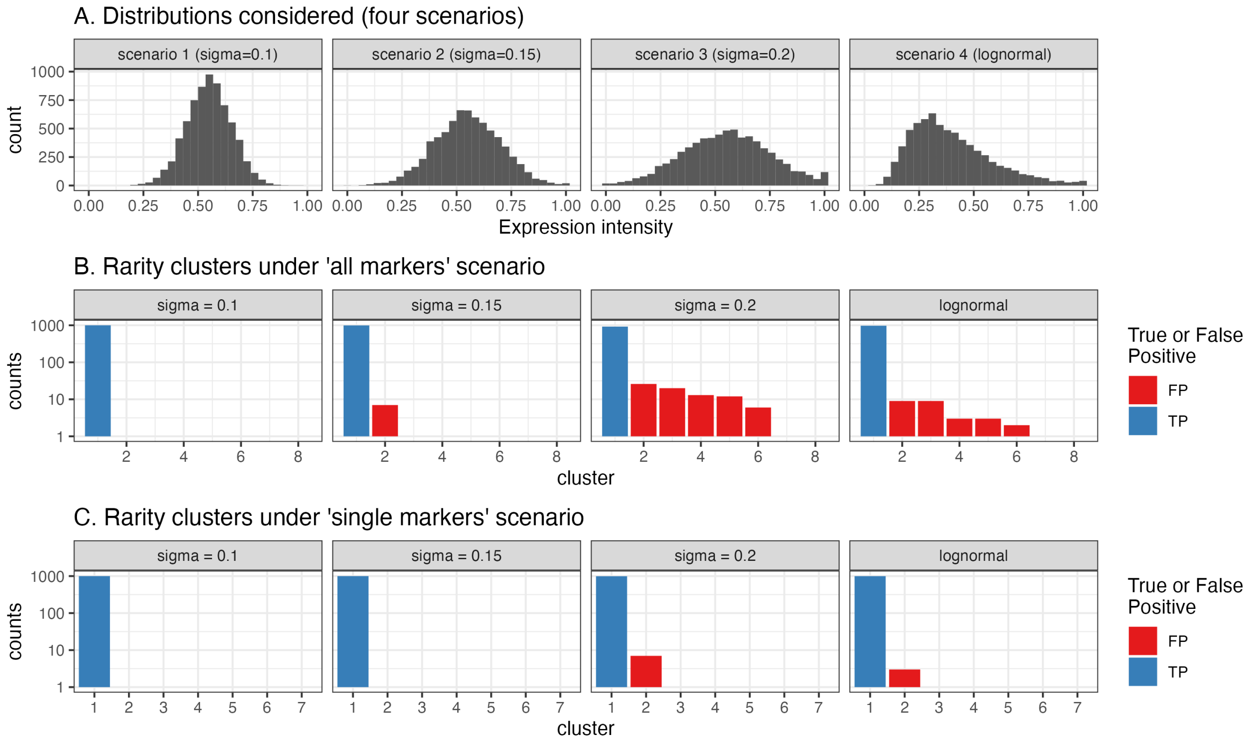


**Supplementary Figure 3:** Simulation study, illustrating the number of clusters (and their sizes) identified by Rarity when ground truth data is generated independently from one of the distributions in panel (A). Under the (B) “all markers” and (C) “single marker” scenario, we show the number of cells (out of 1000) assigned to the ground truth cluster (TP, in blue) and false positive discoveries (FP, in red). Note that the $y$-axis is on log-scale.

These results indicate that while these configurations have substantially different numbers of clusters (and perhaps interestingly, the highest V-measure does not correspond to the configuration with the ground truth number of clusters), the differences in the respective conditional V-measure values are relatively small.

To gain more insight into the identified clusters themselves, we show alluvial plots in Supplementary Figure 5 for the best configurations of PhenoGraph and FlowSOM (for the 0.5% rare cell scenario). PhenoGraph has been quite successful in separating out cell type E, however cell type D has been split across multiple clusters. FlowSOM has embedded cell type E into one larger cluster, and cell types D across multiple clusters. One could further perform meta-clustering on the obtained clusters in Supplementary Figure 5, but this can only merge existing clusters, thus it would not help with separating out the rare cells that have already been embedded into larger clusters.

We conclude that careful hyperparameter tuning, even when we have access to the (conditional) V-measure metric, is not sufficient for identifying the underlying rare groups.

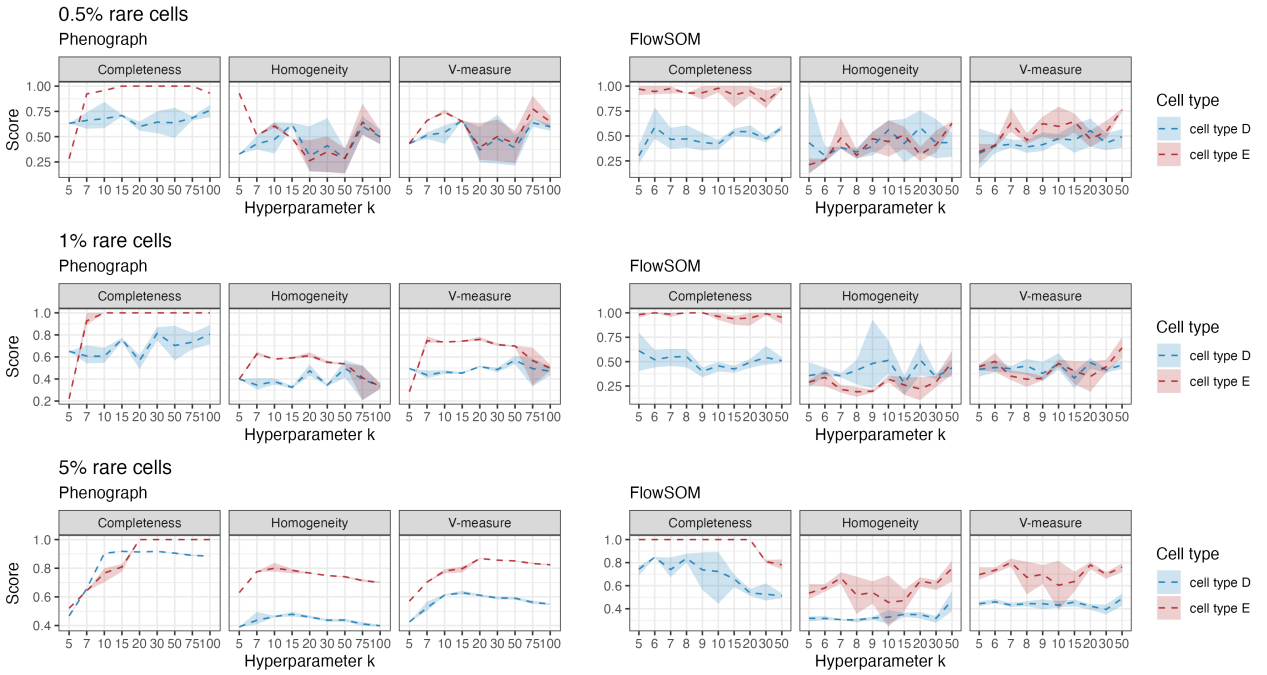


**Supplementary Figure 4:** Varying the hyperparameters of PhenoGraph (left column) and FlowSOM (right column) on the synthetic dataset where ground truth clusters are available. We show conditional completeness, homogeneity and V-measure separately for both rare cell types D and E (shown in colour), across varying proportions of rare cells (0.5%, 1%, 5% in rows).

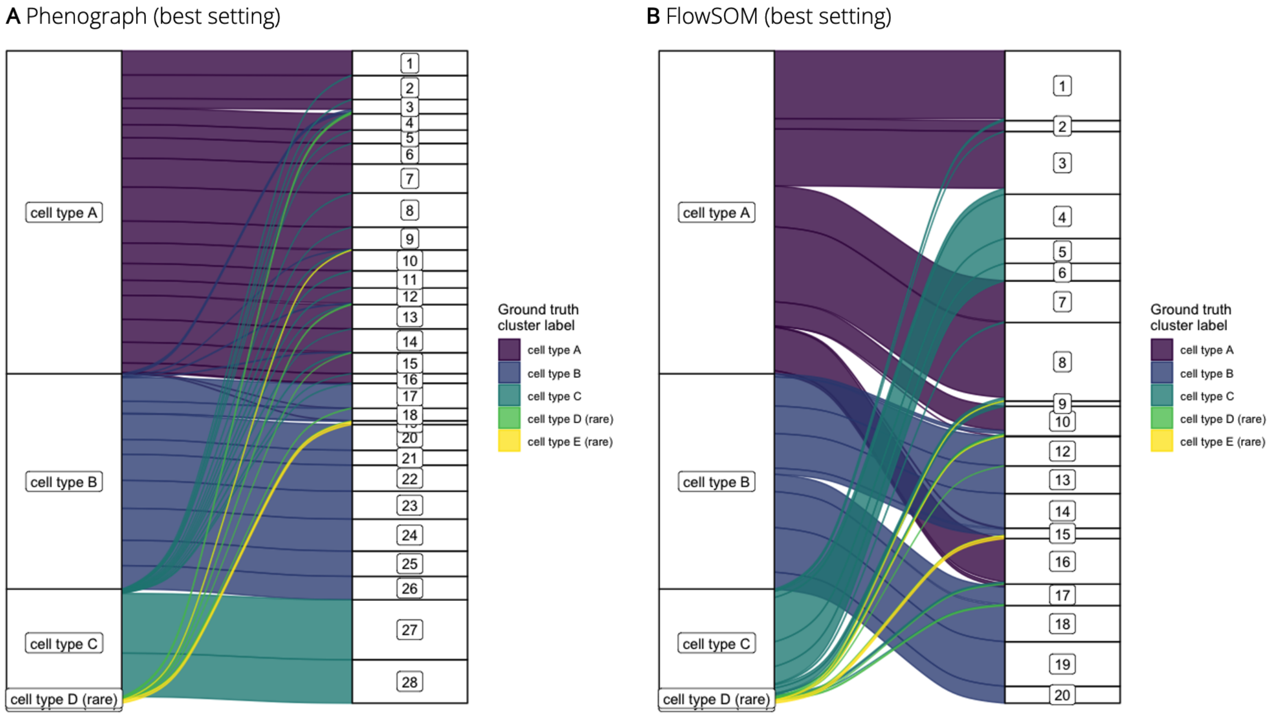


**Supplementary Figure 5:** Illustrating the clustering corresponding to the best setting (obtained from Figure 4, scenario 0.5% rare cells) for both Phenograph and FlowSOM, in comparison to ground truth cluster labels.

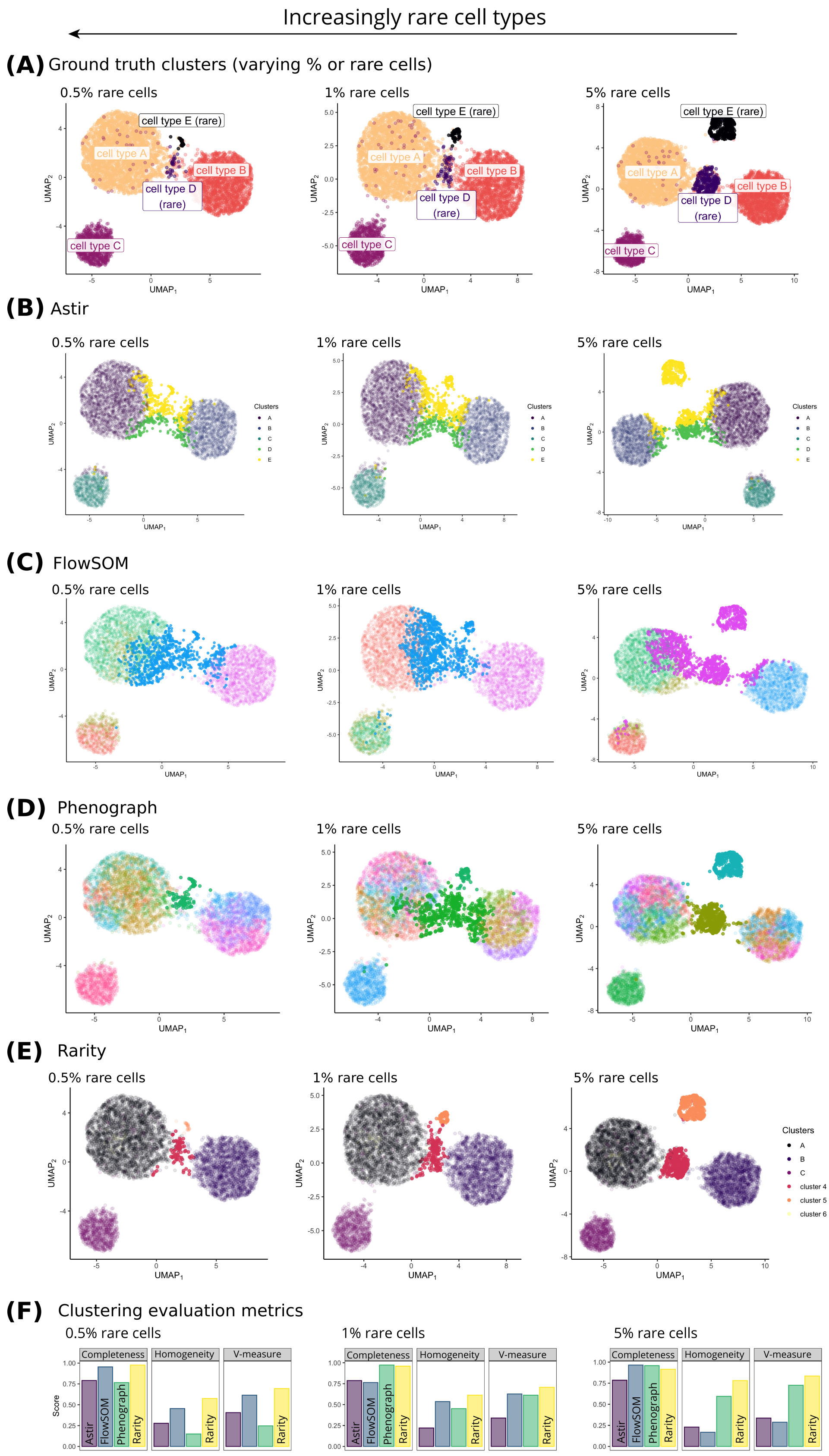


**Supplementary Figure 6:** Extended version of Figure 4, now also including Astir and FlowSOM.

# D Expanded version of Figure 4

# E Meta-clustering does not improve rare cluster identification

In Figure 9E, we include a comparison between Rarity and PhenoGraph on the double-negative T cell example. Here, we expand this comparison, considering PhenoGraph, FlowSOM and PhenoGraph

followed by meta-clustering (using hierarchical clustering with a pre-determined number of meta-clusters) with different hyperparameter values, as shown below in Supplementary Figure 7. In all panels in Supplementary Figure 7, the double-negative T cells have been incorporated as part of various bigger clusters, thus we would not have been able to separate these cells out as a cell population of interest.

## F Sensitivity to cell types that differ by non-binary expression changes

We recognise the limitation of Rarity is that cell types that differ from others only via changes in absolute levels of expression would not be identified.

To explicitly investigate this limitation, we have modified the synthetic data generative mechanism used in Figures 1 and 4, deviating from the assumption that marker genes are either "on" or "off", now allowing differential expression to be a continuous-valued quantity $\Delta$. Specifically, we focus on relaxing this assumption for the marker gene defining cell type E (expression shown in the boxplot in Supplementary Figure 8), where the average intensity of all other cell types is 0.55 but for cell type E it is $(0.55 - \Delta)$ for varying levels of $\Delta$.

Rarity is most successful in detecting cell type E in scenarios where $\Delta$ values are relatively large (0.50 and 0.45), whereas its performance drops significantly for smaller values of $\Delta$ (Supplementary Figure 8). This demonstrates when Rarity's binary expression assumption breaks down - indeed for smaller $\Delta$ values the average expression intensity for cell type E increasingly deviates from zero, thus becoming challenging to distinguish for Rarity. In contrast, the performance of Phenograph holds more firmly for smaller $\Delta$ values. However, this sensitivity is gained through a corresponding increase in the number of false cell types. In this example, Phenograph identifies a total number of 15 clusters, subdividing the 5 true cell types into further subtypes based on expression heterogeneity within each cell type.

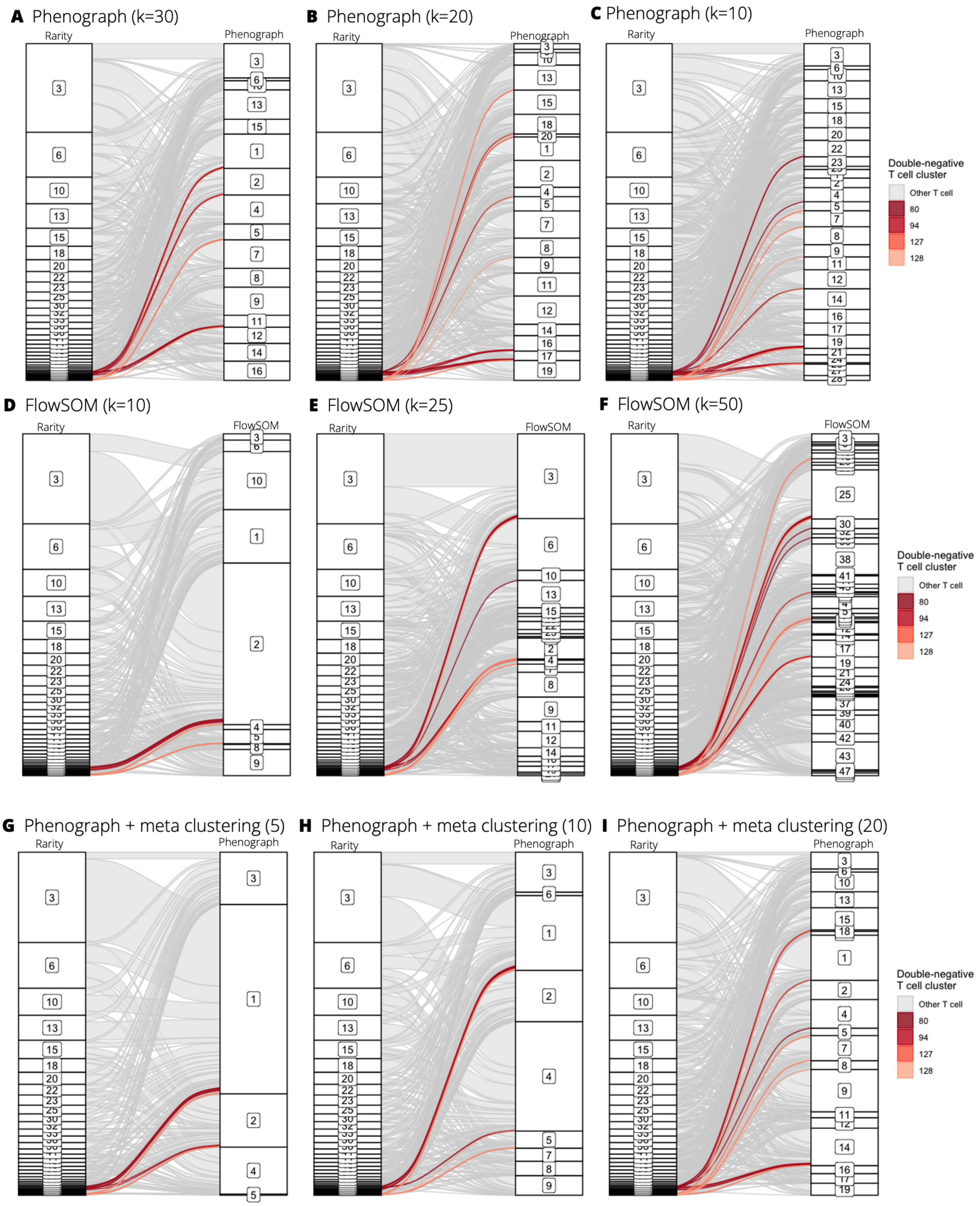


**Supplementary Figure 7:** Double-negative T cell example revisited: Comparing Phenograph (with varying number of nearest neigbours), FlowSOM (with varying number of clusters), and Phenograph with meta-clustering (where meta-clustering was performed using hierarchical clustering on PhenoGraph cluster centroids, with a varying number of meta-clusters) on the double-negative T cell example.

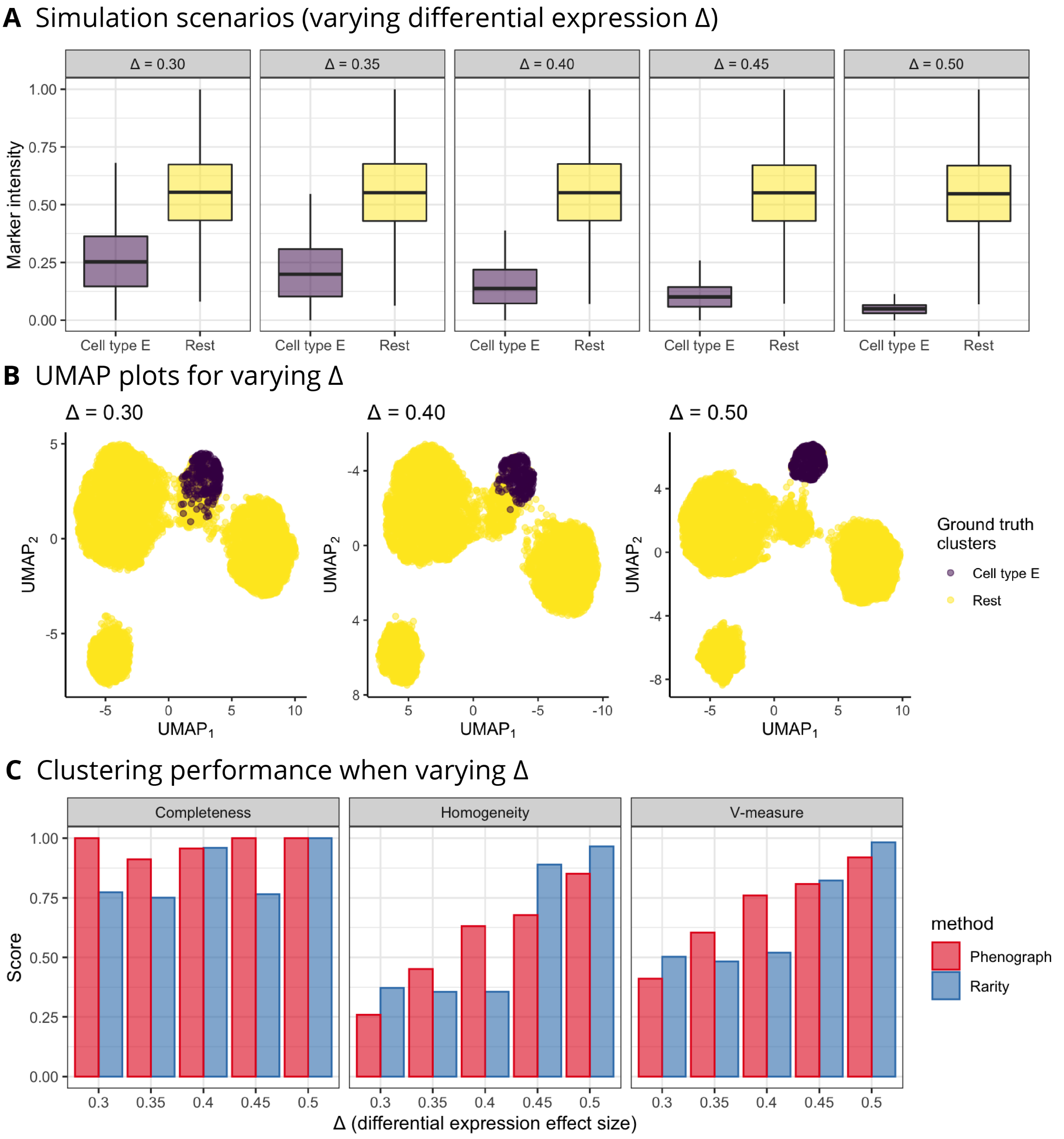


**Supplementary Figure 8:** Limitations of Rarity: challenging the assumption that marker genes are either "on" or "off". Considering a synthetic data generative mechanism similar to Figures 1 and 4, here we demonstrate what happens when the discriminative marker for cell type E exhibits expression intensities decreased by amount $\Delta$ for varying levels of $\Delta$. Specifically, for the marker shown in (A), the average intensity of all other cell types is 0.55 whereas for cell type E it is (0.55-$\Delta$) for varying levels of $\Delta$. The respective UMAP visualisations are shown in (B). Rarity is most successful in detecting cell type E in scenarios where $\Delta$ values are relatively large (0.50 and 0.45), as shown in panel (C), whereas its performance drops significantly for smaller values of $\Delta$. This illustrates when Rarity's binary expression assumption breaks down - indeed for smaller values the average expression intensity for cell type E starts to deviate far from zero, thus becoming challenging to distinguish for Rarity. This is in contrast to Phenograph (C) whose performance also decreases for smaller $\Delta$ values, but its performance fades more slowly as Phenograph does not take into account the absolute levels of expression.

**Table 1:** Classification perspective: Treating PhenoGraph output as a classifier, where all clusters with less than 2% of cells are classified as "rare". Those clusters containing a majority of rare cell type D (or E) are classified as true positives (TP), the rest as false positives (FP). An empirical estimate of the positive predictive value (PPV) is also shown.

| | | $k = 5$ | $k = 7$ | $k = 10$ | $k = 15$ | $k = 20$ | $k = 30$ |
|---|---|---|---|---|---|---|---|
| rare cell type D | # TP | 0 | 0 | 0 | 0 | 0 | 0 |
| | # FP | 68 | 27 | 7 | 2 | 2 | 1 |
| | PPV | 0.0 | 0.0 | 0.0 | 0.0 | 0.0 | 0.0 |
| rare cell type E | # TP | 0 | 1 | 1 | 1 | 1 | 1 |
| | # FP | 68 | 26 | 6 | 1 | 1 | 0 |
| | PPV | 0.0 | 0.04 | 0.14 | 0.50 | 0.50 | 1.0 |

# G Classification metrics are not suitable for performance benchmarking

To take the classification perspective here, we would first need to introduce a classification rule, when to call a PhenoGraph cluster true positive and when a false positive. For this simulation experiment, using our first synthetic dataset with rare cell types D and E, we define all PhenoGraph clusters which contain less than 2% of cells as "rare". Those clusters truly containing a majority of rare cell type D (or E) are classified as true positives (TP), the rest as false positives (FP). This lets us empirically estimate the positive predictive value (PPV) that a given PhenoGraph rare cluster substantially overlaps with rare cell types of interest. Table 1 shows that with $k = 5$ nearest neighbours, we detect 68 rare clusters, but none of them has a majority cell type D or E. With $k = 10$, PhenoGraph does identify a cluster that contains rare cells E (and 6 other false positives, which gradually disappear when increasing $k$ further, leading to increasing PPV values). However cell type D is missed throughout. We recognise that instead of defining true and false positives based on majority cells, other decision thresholds could equally be considered to get a more in-depth understanding.

Now if we wanted to present an analogous analysis for Rarity, it would be important to incorporate the fact that Rarity clusters come with interpretable expression signatures, but it is unclear to us how to do this in the binary classification framework. When naively counting clusters, as shown in the original Figure 1D, Rarity identifies four rare clusters: one for cell type D, one for cell type E, and two additional clusters. Then for cell type D, then TP=1 and FP=3, and for cell type E also TP=1 and FP=3.

To further highlight the difference between PhenoGraph and Rarity clusters, we now show the heatmap of PhenoGraph ($k = 10$) clusters (analogous to the original Figure 1D) in Supplementary Figure 9. All rare clusters (i.e. those with prevalence below 2%) are shown in Supplementary Figure 9 panel B. This complements the above TP and FP counts, illustrating how rare cell type D ends up being mixed into clusters 15 and 18.

Overall, while classification metrics would indeed be more canonical, we do not think they are best suited here to characterise rare cluster detection performance due to the fact that there is significant reconfiguration of clustering structure with hyper-parameter changes. Further, the terminology of binary classification is not straightforward to adapt to distinguish between the scenarios where truly rare cell populations are embedded within bigger clusters or separated into multiple sub-clusters. This is why we adopted the suggested metrics.

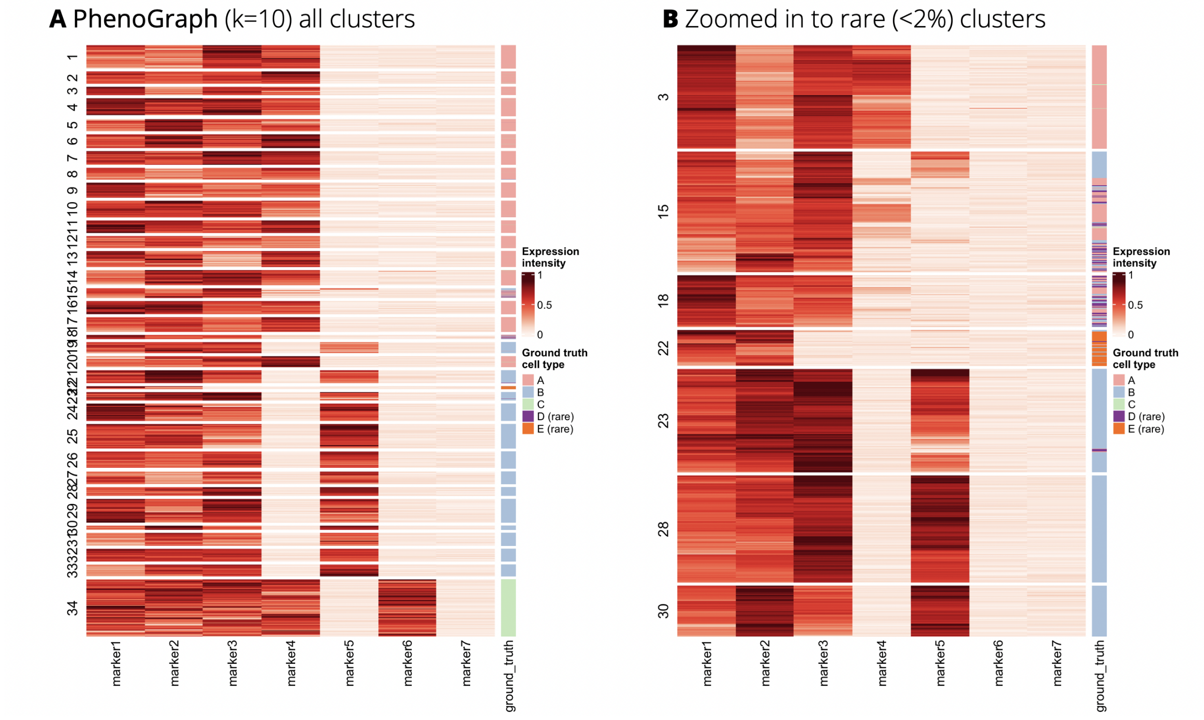


**Supplementary Figure 9:** Heatmap illustrating PhenoGraph ($k = 10$) clusters on the synthetic example. Heatmap rows are split according to PhenoGraph clusters, and vertical annotation bar (five colours) show ground truth clusters. (A) shows all PhenoGraph clusters, and (B) is a zoomed in version where only rare clusters (with less than 2% prevalence) have been shown. Cluster number 22 is counted as a true positive for cell type E.

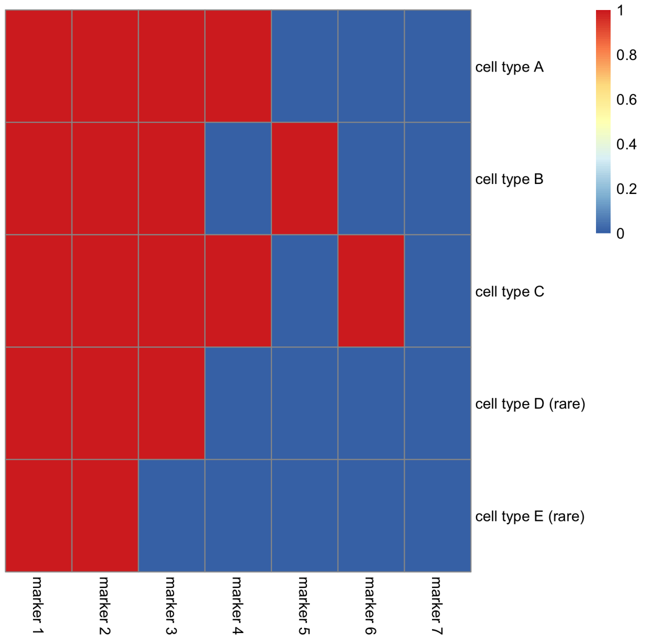


**Supplementary Figure 10:** Marker genes illustrated for the synthetic data used in the main Figures 1 and 4.

# H Synthetic example: marker genes

# I Additional scenario: a rare cell type with many marker genes

To explicitly demonstrate that a larger number of differentially expressed genes would make it much easier to detect rare cell types, we have expanded this example by including a third rare cell type F which differentially expresses 4 additional markers.

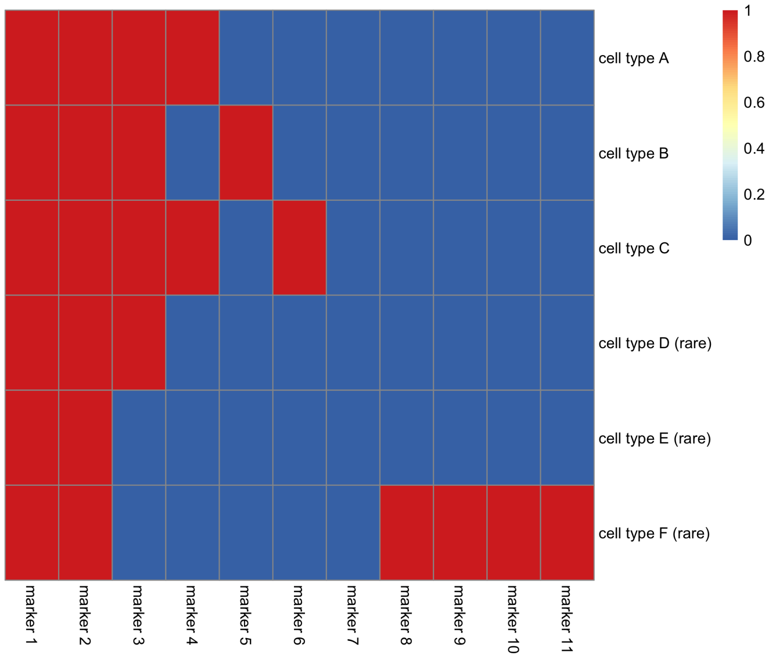


**Supplementary Figure 11:** Marker genes illustrated for the additional scenario where we include an additional rare cell type F that is defined by relatively many marker genes.

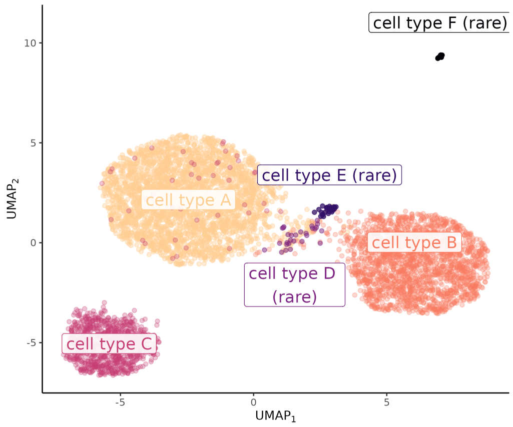


**Supplementary Figure 12:** UMAP plot illustrating the cell landscape, now including the additional rare cell type F.

The resulting dataset has been illustrated in 2D using UMAP. We compare all clustering methods and summarise their performance with the conditional V-measure for every rare cell type separately in the Table below. In this scenario, cell type F is much easier to detect than cell types D and E. Indeed, all methods achieve perfect conditional V-measure metric 1.0 for cell type F, despite the low 0.5% prevalence.

**Table 2:** Conditional V-measure shown for all rare cell types (D, E, F) across various clustering methods (FlowSOM, Phenograph, Astir, Rarity).

| | FlowSOM | Phenograph | Astir | Rarity |
|---|---|---|---|---|
| cell type D | 0.48 | 0.39 | 0.54 | 0.54 |
| cell type E | 0.35 | 0.40 | 0.47 | 0.88 |
| cell type F | 1.00 | 1.00 | 1.00 | 1.00 |
