# Supplementary material for: Rarity: discovering rare cell populations from single-cell imaging data
Source: Bioinformatics. 2023 Dec 13;39(12):btad750. doi: 10.1093/bioinformatics/btad750 (PMC10751233; doi:10.1093/bioinformatics/btad750)
Supplement: btad750_Supplementary_Data [file btad750_supplementary_data.zip › supp-tables.pdf]

Supplementary Tables

Supplementary Table 3. Samples used in the study

For each sample reported are the ID, the tissue and anatomical site of origin, the source of the sample and the figure where these data are shown. UCLH: University College London Hospital, ICH: Istituto Clinico Humanitas, NA: not available.

Sample ID	Tissue	Anatomical site	Source	Reference
soCRC_1	Human colon mucosa	NA	UCLH	Fig.9,10
soCRC_2	Human colon mucosa	NA	UCLH	Fig.9,10
soCRC_3	Human colon mucosa	NA	UCLH	Fig.9,10
soCRC_4	Human colon mucosa	NA	UCLH	Fig.9,10
soCRC_5	Human colon mucosa	NA	UCLH	Fig.9,10
soCRC_6	Human colon mucosa	NA	UCLH	Fig.9,10
syCRC_1	Human colon mucosa	Rectum	UCLH	Fig.9,10
syCRC_2	Human colon mucosa	Transverse colon	UCLH	Fig.9,10
syCRC_3	Human colon mucosa	Ascending colon	UCLH	Fig.9,10
syCRC_4	Human colon mucosa	Descending colon	UCLH	Fig.9,10
syCRC_5	Human colon mucosa	Rectum	UCLH	Fig.9,10
syCRC_6	Human colon mucosa	Ascending colon	ICH	Fig.9,10
syCRC_7	Human colon mucosa	Ascending colon	ICH	Fig.9,10
syCRC_8	Human colon mucosa	Descending colon	ICH	Fig.9,10
syCRC_9	Human colon mucosa	Descending colon	ICH	Fig.9,10
syCRC_10	Human colon mucosa	Ascending colon	ICH	Fig.9,10

Supplementary Table 4. Antibodies used in the study

For each antibody reported are the associated cell population, the catalogue number, the vendor, the tag, the dilution used in the staining. Data shown in figures 5, 6, and 7 were derived from a previously published breast cancer study(Jackson et al. 2020).

Cell population	Antibody Specificity	Vendor	Catalogue Number	Metal Tag	Reference
All leukocytes	CD45	Fluidigm	3152016D	152Sm	Fig.9,10

B cells	CD20	Fluidigm	3161029D	161Dy	Fig.9,10
B cells	IgA	NovusBio	NB500-469	142Nd	Fig.9,10
B cells	IgM	NovusBio	NBP2-34254	169Tm	Fig.9,10
B cells / T cells	CD27	Fluidigm	3171024D	171Yb	Fig.9,10
T cells	CD45RA	Fluidigm	3166028D	166Er	Fig.9,10
T cells	CD45RO	Fluidigm	3173016D	173Yb	Fig.9,10
T cells / macrophages	CD4	Fluidigm	3156033D	156Gd	Fig.9,10
T cells	CD8	Fluidigm	3162035D	162Dy	Fig.9,10
T cells	PD1	Fluidigm	3165039D	165Ho	Fig.9,10
T cells	CD3	Fluidigm	3170019D	170Er	Fig.9,10
T cells	FOXP3	Fluidigm	3155016D	155Gd	Fig.9,10
Macrophages	CD68	Fluidigm	3159035D	159Tb	Fig.9,10
Macrophages	CD16	Fluidigm	3146020D	146Nd	Fig.9,10
Macrophages and dendritic cells	CD11c	Abcam	ab216655	175 Lu	Fig.9,10
Macrophages, dendritic cells, tumour cells	PDL1	RnD System	MAB1561	150Nd	Fig.9,10
Endothelial cells	CD34	Abcam	ab213058	164Dy	Fig.9,10
Epithelial cells	Pan keratin	Fluidigm	3148020D	148Nd	Fig.9,10
Epithelial cells	E-Cadherin	Fluidigm	3158029D	158Gd	Fig.9,10
Basement membrane cells	Collagen type IV	NovusBio	NBP1-97716	176Yb	Fig.9,10
Proliferating cells	Ki67	Fluidigm	3168022D	168Er	Fig.9,10
Stromal cells	Vimentin	Fluidigm	3143029D	143Nd	Fig.9,10
Stromal cells	SMA	Fluidigm	3141017D	141Pr	Fig.9,10
Various	CAMK4	NovusBio	NBP2-37428	174Yb	Fig.9,10
Various	IFNA5	CloudClone	MAG975Hu22	147Sm	Fig.9,10
Various	VEGFC	Abcam	ab191274	154Sm	Fig.9,10
All Nuclei	H3	Cell Signaling	4499BF	In113	Fig.5,6,7

Various	H3K9me3	Cell Signaling	9733BF	La139	Fig.5,6,7
Basal epithelium	CK5	Abcam	Custom	Pr141	Fig.5,6,7
Stromal cells	Fibronectin	BD Biosciences	610078	Nd142	Fig.5,6,7
Luminal epithelium	CK19	Dev Studies Hybridoma Bank Troma-III	37815	Nd143	Fig.5,6,7
Luminal epithelium	CK8/18	Cell Signaling	4546BF	Nd144	Fig.5,6,7
Various	Twist	Millipore	ABD29	Nd145	Fig.5,6,7
Macrophages	CD68	E-Bioscience	14-0688-82	Nd146	Fig.5,6,7
Basal epithelium	KRT14	Thermo Fischer	PA5-16722	Sm147	Fig.5,6,7
Stromal cells	SMA	Abcam	ab7817	Nd148	Fig.5,6,7
Stromal cells	Vimentin	Cell Signaling	5741BF	Sm149	Fig.5,6,7
Various	c-Myc	Biolegend	626802	Nd150	Fig.5,6,7
Her2 cancer	HER2	BD Biosciences	554299	Eu151	Fig.5,6,7
T Cells	CD3ε	Cell Signaling	85061	Sm152	Fig.5,6,7
All Nuclei	H3	Biolegend	641002	Eu153	Fig.5,6,7
Various	Slug	R&D Systems	Custom	Gd155	Fig.5,6,7

ERα+ cancer	ERα	Epitomics	AC-0015EU	Gd156	Fig.5,6,7
PR+ cancer	PR A/B	Spring Bioscience	M3024 C	Gd158	Fig.5,6,7
PR+ cancer	PR A/B	Epitomics	AC-0028EU	Gd158	Fig.5,6,7
All cells	p53	Cell Signaling	2527BF	Tb159	Fig.5,6,7
Various	CD44	R&D Systems	AF3660	Gd160	Fig.5,6,7
All leukocytes	CD45	E-Bioscience	14-9457-82	Dy162	Fig.5,6,7
Epithelilal cells	GATA3	BD Biosceinces	558686	Dy163	Fig.5,6,7
B cells	CD20	E-Bioscience	14-0202-82	Dy164	Fig.5,6,7
Various	CA9	R&D Systems	AF2188	Er166	Fig.5,6,7
Epithelilal cells	E-Cadherin/P-Cadherin	BD Biosciences	610182	Er167	Fig.5,6,7
Proliferating cells	Ki67	Cell Signaling	9449BF	Er168	Fig.5,6,7
EGFR+ cancer	EGFR	Cell Signaling	4267BF	Tm169	Fig.5,6,7
Various	p-S6	Cell Signaling	4858BF	Yb170	Fig.5,6,7
Various	vWF	Millipore	AB7356	Yb172	Fig.5,6,7
Endothelial cells	CD31	Novus Biologicals	NB600-562	Yb172	Fig.5,6,7
Various	p-mTOR	Cell Signaling	2976	Yb173	Fig.5,6,7

Luminal epithelium	CK7	Biosciences	550507	Yb174	Fig.5,6,7
Epithelilal cells	Pan CK	MAB1612	2341224	Lu175	Fig.5,6,7
Epithelilal cells	Pan CK	MAB1611	2607604	Lu175	Fig.5,6,7
Apoptosis	cleaved PARP	BD Biosciences	552596	Yb176	Fig.5,6,7
Apoptosis	Cleaved Caspase3	BD Biosciences	559565	Yb176	Fig.5,6,7

Supplementary Table 5. Cell type definition for epithelial luminal cells (for Figure 6)

We defined epithelial luminal cells as those expressing the following markers:

- DNA1, DNA2
- E-cadherin
- Pan-Cytokeratin
- Cytokeratin 7
- Cytokeratin 8/18
- Cytokeratin 19

whereas not expressing the following markers

- Vimentin
- vWF
- SMA
- CD45
- CD3
- CD20
- CD68

Supplementary Table 6. Cell type definitions for colon mucosa data (used in Figure 9A)

	CD45	CD3	CD68	CD20	IgA	CD11c	CD34	SMA	eCadherin	CollagenIV
T cells	1	1	0	0						
B cells	1	0	0	One of CD20/IgA						
Macrophages	1	0	1	0						

Dendritic cells	0					1		0		
Epithelial	0								1	
Endotheli al	0						1			
Connectiv e	0							1		1
